# Supplementary material for: Carbenoid-involved reactions integrated with scaffold-based screening generates a Nav1.7 inhibitor
Source: Commun Chem. 2024 Jun 12;7:135. doi: 10.1038/s42004-024-01213-3 (PMC11169417; doi:10.1038/s42004-024-01213-3)
Supplement: Supplementary file 2 — Supplementary Information [file 42004_2024_1213_MOESM2_ESM.pdf]

Supplementary Information for

Carbenoid-involved Reactions Integrated with Scaffold-based Screening Generates a Nav1.7 inhibitor

Jirong Shu[1, ‡], Yuwei Wang [2, ‡] , Weijie Guo [2], Tao Liu [2] , Song Cai [2,*], Taoda Shi [1 ,*], Wenhao Hu[1 ,*].

[1] School of Pharmaceutical Sciences, Sun Yat-sen University, Guangzhou, China, 510006.
[2] Shenzhen University Health Science Center, Shenzhen 518060, China.
[‡] These authors contributed equally: Jirong Shu, Yuwei Wang
[*]These authors jointly supervised this work: Song Cai (caisong@szu.edu.cn); Taoda Shi (shitd@mail.sysu.edu.cn); Wenhao Hu (huwh9@mail.sysu.edu.cn)

This PDF file includes:

Sections S1 to S9
Supplementary figure 1 to 12
Supplementary Tables. 1 to 5
Supplementary References

1. Supplementary information of virtual compound library

Supplementary Table 1. Reaction list of compound library

Reaction	No.	Ref
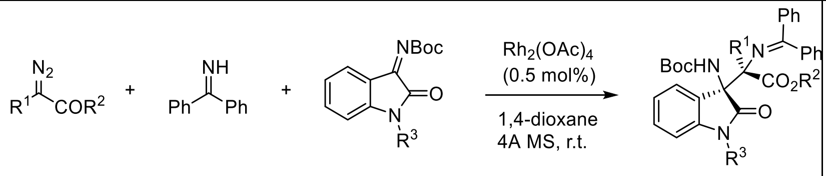 i-1-a1 i-1-b i-1-c i-1-d	Reaction_i-1-1	1
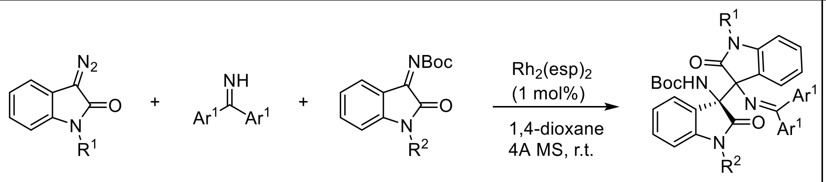 i-1-a2 i-1-b i-1-c i-1-d	Reaction_i-1-2	1
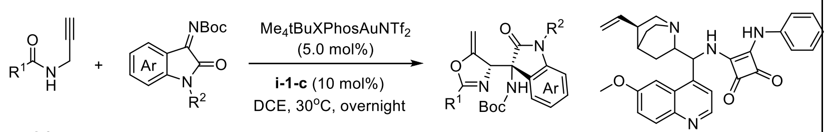 **i-2-a** **i-2-b1** **i-2-e1** **i-2-c**	Reaction_i-2-1	2
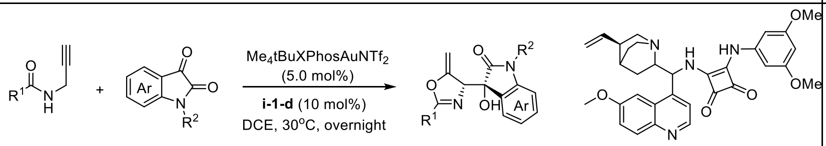 **i-2-a** **i-2-b2** **i-2-e2** **i-2-d**	Reaction_i-2-2	2
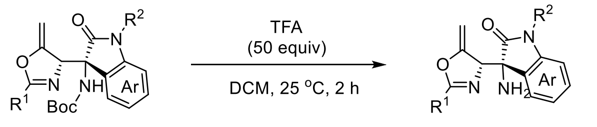 **i-2-e1** **i-2-f**	Reaction_i-2-3	2
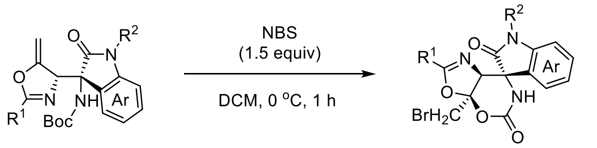 **i-2-e1** **i-2-g**	Reaction_i-2-4	2
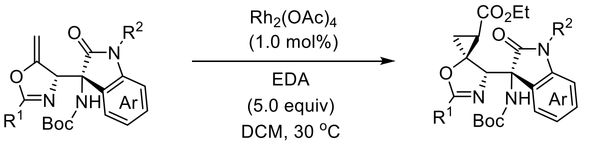 **i-2-e1** **i-2-h**	Reaction_i-2-5	2
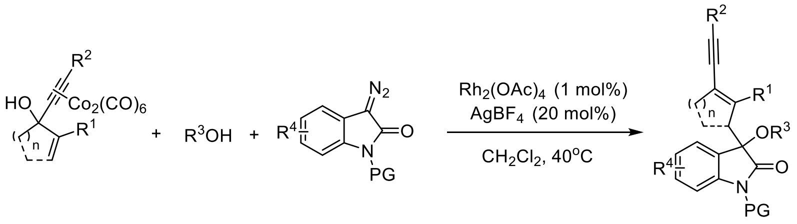 **i-3-a** **i-3-b** **i-3-c** **i-3-d**	Reaction_i-3	3

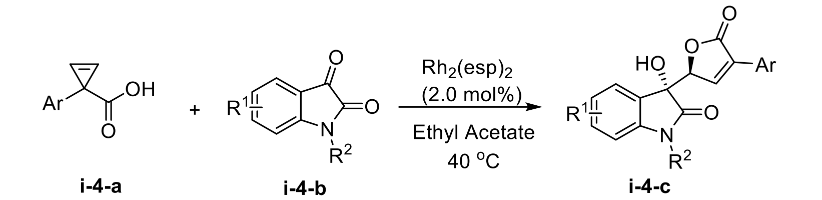	Reaction_i-4	4
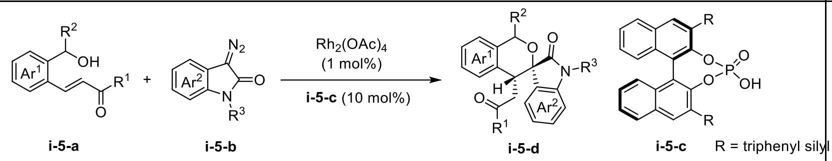	Reaction_i-5-1	5
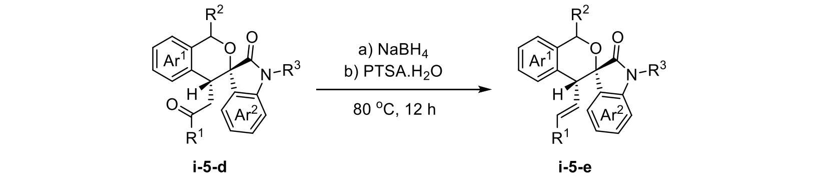	Reaction_i-5-2	5
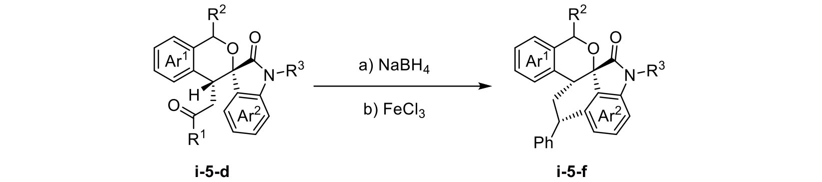	Reaction_i-5-3	5
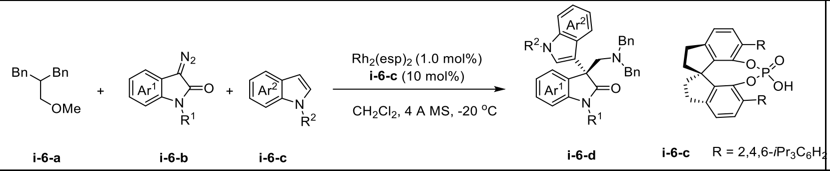	Reaction_i-6-1	6
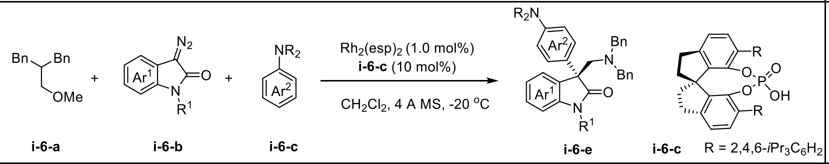	Reaction_i-6-2	6
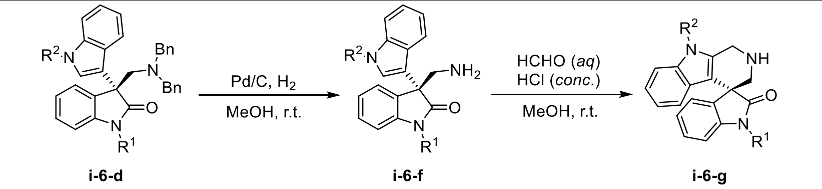	Reaction_i-6-3	6
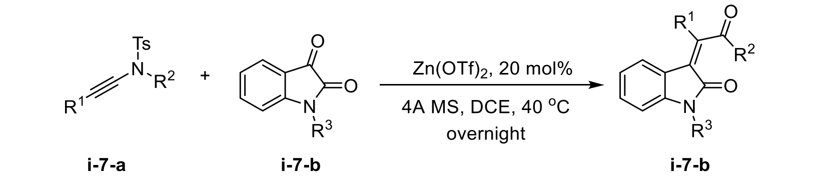	Reaction_i-7	7
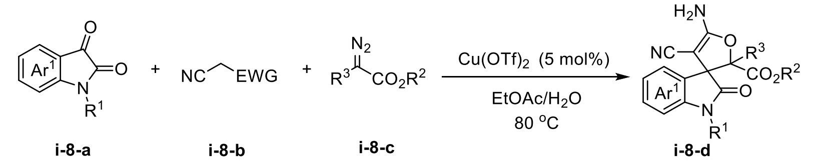	Reaction_i-8	8
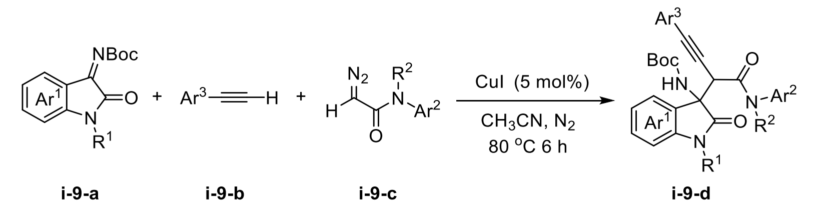	Reaction_i-9	9

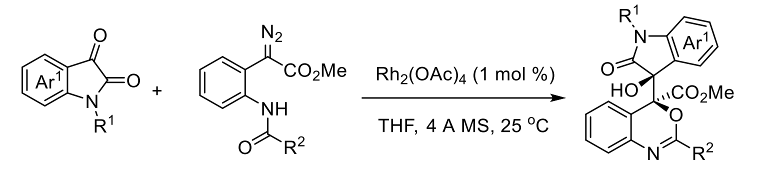 i-10-a i-10-b i-10-c	Reaction_i-10	10
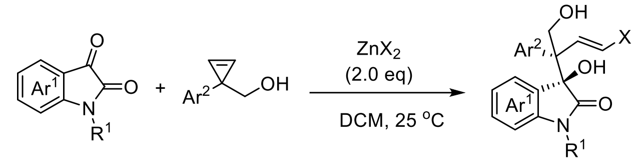 i-11-a i-11-b i-11-c	Reaction_i-11-1	11
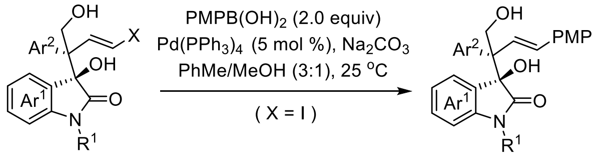 i-11-c i-11-d	Reaction_i-11-2	11
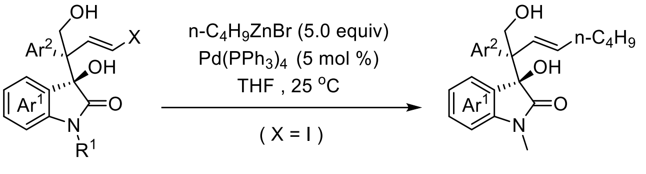 i-11-c i-11-e	Reaction_i-11-3	11
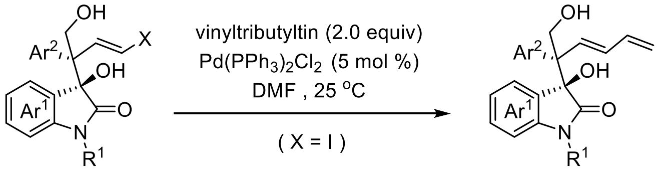 i-11-c i-11-f	Reaction_i-11-4	11
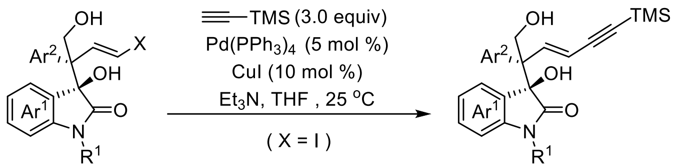 i-11-c i-11-g	Reaction_i-11-5	11
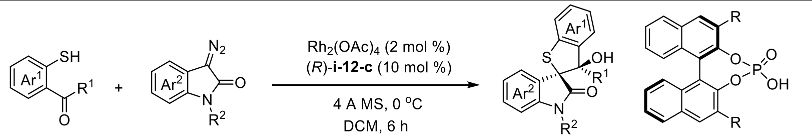 i-12-a i-12-b i-12-d i-12-c R = SiPh3	Reaction_i-12	12
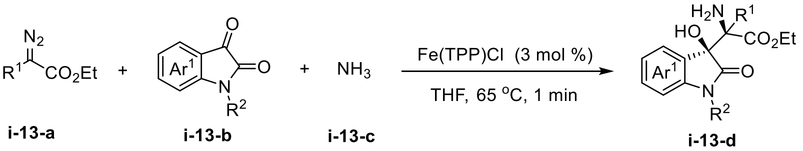 i-13-a i-13-b i-13-c i-13-d	Reaction_i-13-1	13
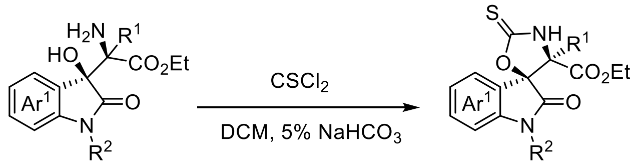 i-13-d i-13-f	Reaction_i-13-2	13

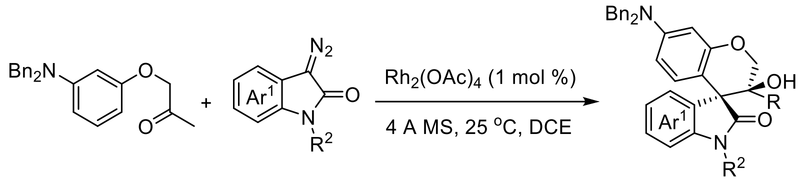<br>i-14-a i-14-b i-14-c	Reaction_i-14	14
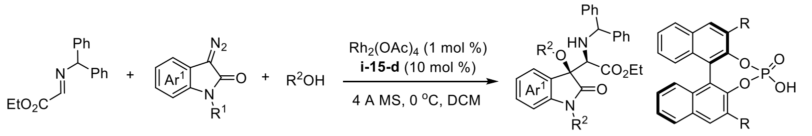<br>i-15-a i-15-b i-15-c i-15-e i-15-d<br>R = 2,4,6-tripropylPh	Reaction_i-15	15
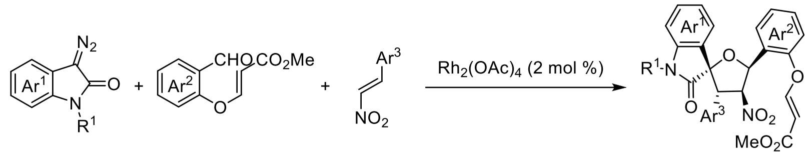<br>i-16-a i-16-b i-16-c i-16-d	Reaction_i-16-1	16
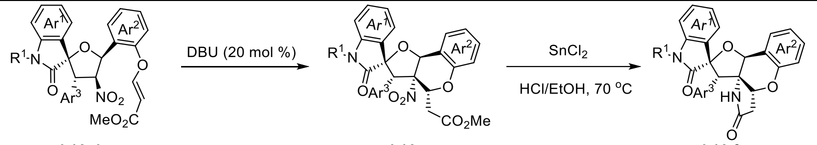<br>i-16-d i-16-e i-16-f	Reaction_i-16-2	16
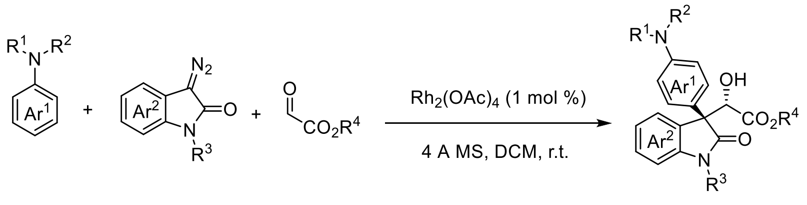<br>i-17-a i-17-b i-17-c i-17-d	Reaction_i-17	17
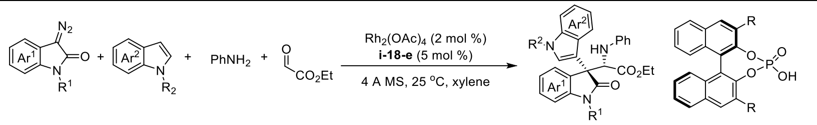<br>i-18-a i-18-b i-18-c i-18-d i-18-f i-18-e R = $SiPh_3$	Reaction_i-18	18
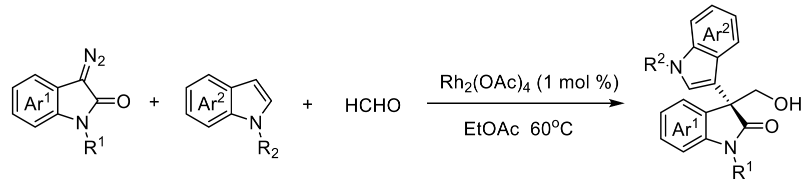<br>i-19-a i-19-b i-19-c i-19-d	Reaction_i-19	19
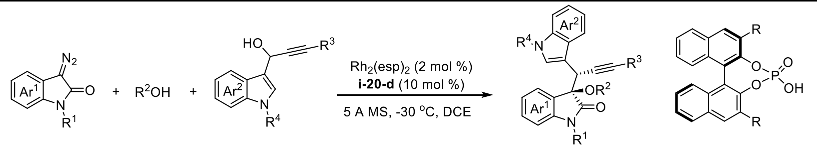<br>i-20-a i-20-b i-20-c i-20-d<br>R = biphenyl	Reaction_i-20-1	20
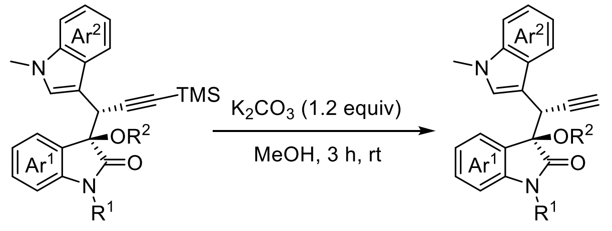<br>i-20-e i-20-f	Reaction_i-20-2	20

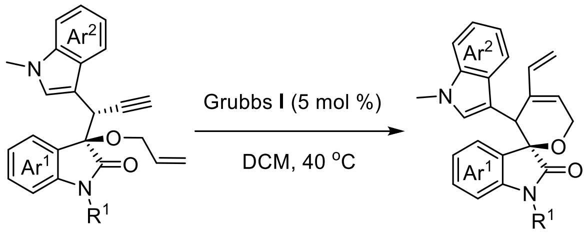 **i-20-f1** R^2 = allyl **i-20-g**	Reaction_i-20-3	20
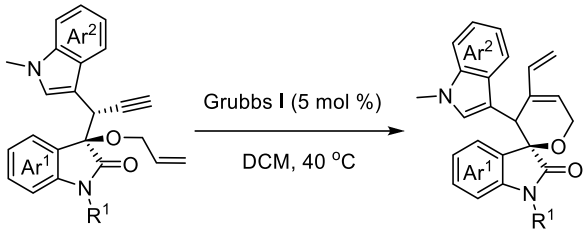 **i-20-f1** R^2 = allyl **i-20-g**	Reaction_i-20-4	20
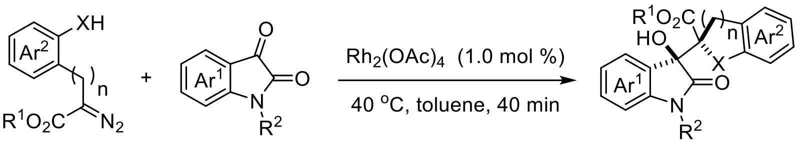 **i-21-a** **i-21-b** **i-21-c**	Reaction_i-21	21
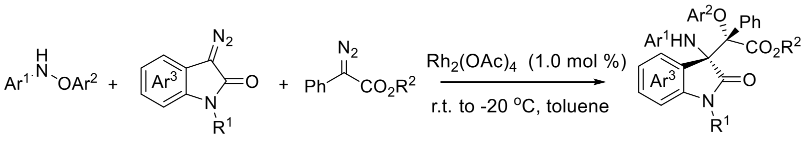 **i-22-a** **i-22-b** **i-22-c** **i-22-d**	Reaction_i-22	22

2.Compounds information

New synthesis compounds

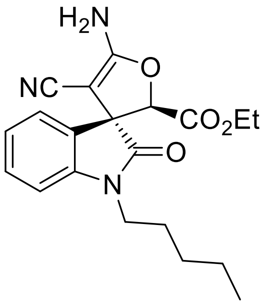

Column chromatography afforded the desired product A3 in 18.3 % yield. as colorless solid. ethyl (2R,3R)-5-amino-4-cyano-2'-oxo-1'-pentyl-2H-spiro[furan-3,3'-indoline]-2-carboxylate.

Trans(A3): ^{1}H NMR (500 MHz, DMSO-*d6*) δ 7.81 (s, 1H), 7.35 (td, *J* = 7.7, 1.5 Hz, 1H), 7.14 – 6.99 (m, 2H), 5.29 (s, 1H), 3.80 – 3.60 (m, 2H), 1.60 (d, *J* = 8.5 Hz, 1H), 1.29 (pd, *J* = 7.5, 6.7, 3.6 Hz, 2H), 0.84 (t, *J* = 6.8 Hz, 2H), 0.56 (t, *J* = 7.1 Hz, 2H).

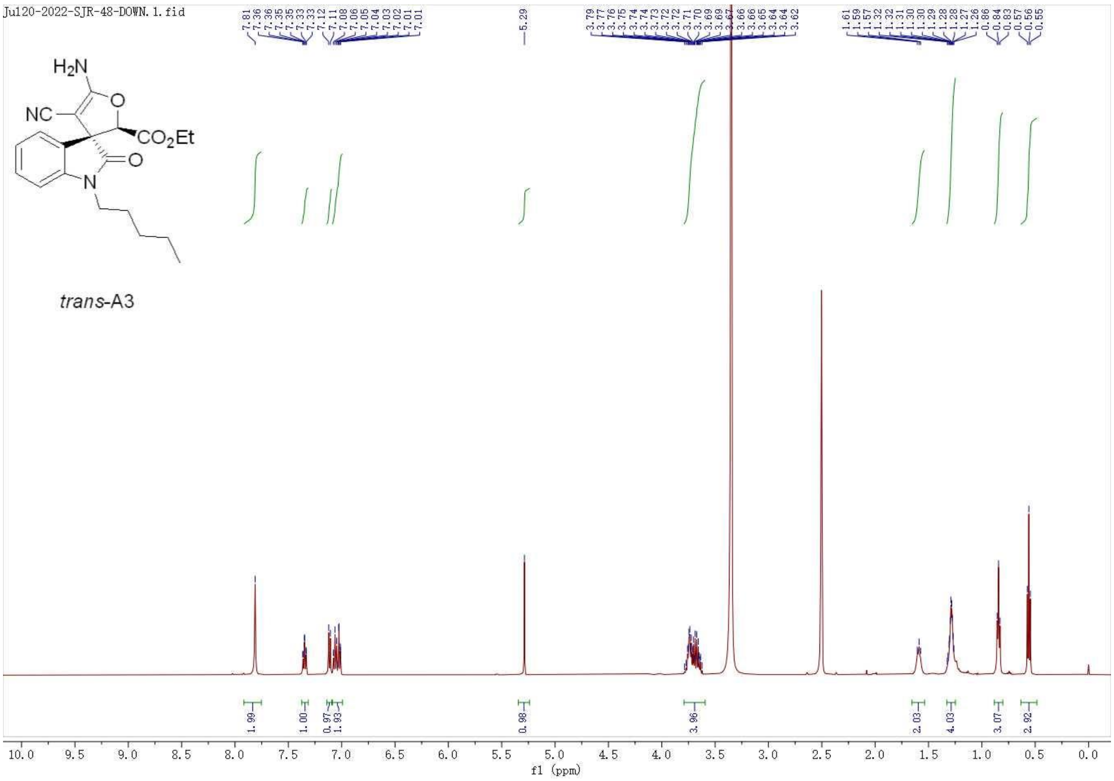

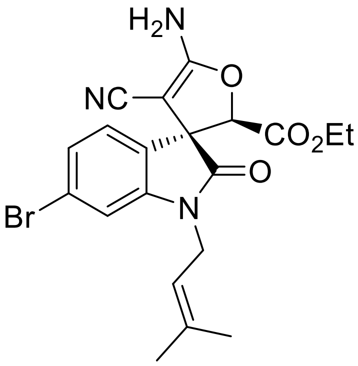

Column chromatography afforded the desired product A4 in 21.3 % yield. as colorless solid

ethyl 5-amino-6'-bromo-4-cyano-1'-(3-methylbut-2-en-1-yl)-2'-oxo-2H-spiro[furan-3,3'-indoline]-2-carboxylate.

Cis(A4): ^{1}H NMR (500 MHz, DMSO-d_6) δ 7.89 (s, 2H), 7.27 (dd, *J* = 7.9, 1.7 Hz, 1H), 7.23 (d, *J* = 1.8 Hz, 1H), 6.96 (d, *J* =

7.9 Hz, 1H), 5.31 (s, 1H), 5.13 – 5.07 (m, 1H), 4.35 (qd, *J* = 15.4, 6.8 Hz, 2H), 3.80 – 3.65 (m, 2H), 1.79 (d, *J* = 1.3 Hz, 3H), 1.68 (d, *J* = 1.5 Hz, 3H), 0.63 (t, *J* = 7.1 Hz, 3H).

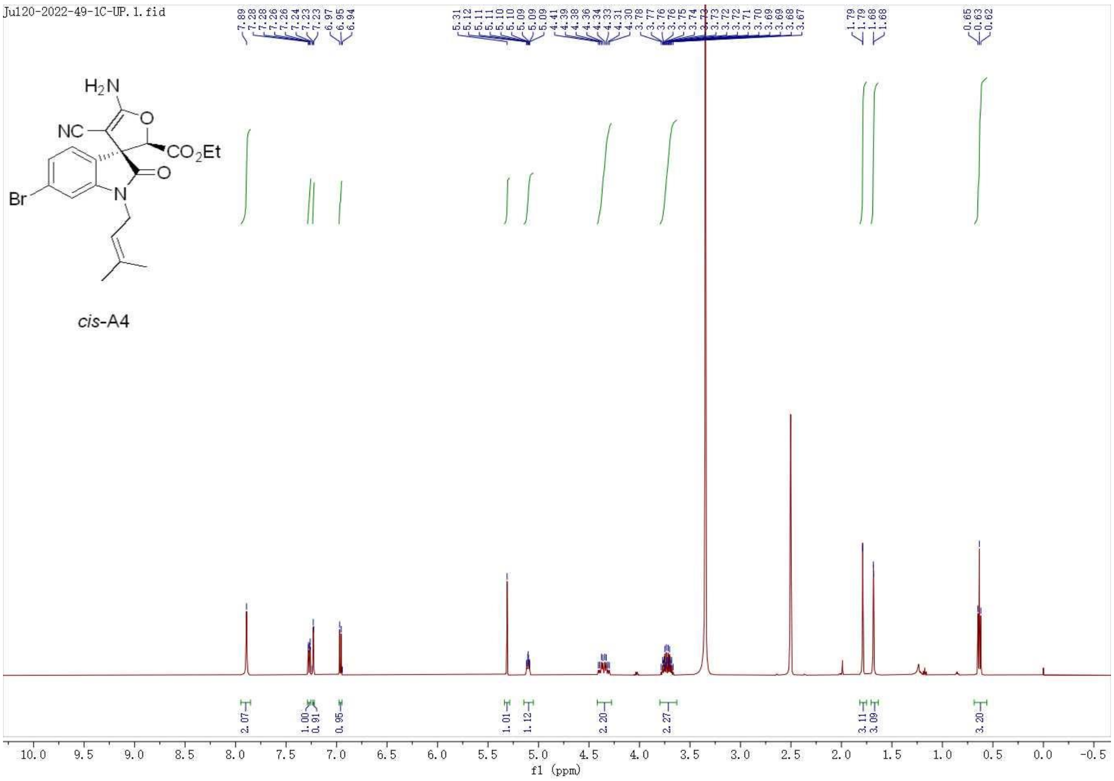

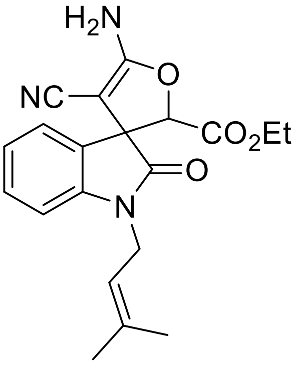

Column chromatography afforded the desired product A5+A6 in 76% yield as colorless solid, cis(A6) : trans(A5) = 42 :58

cis-ethyl (2R,3R)-5-amino-4-cyano-1'-(3-methylbut-2-en-1-yl)-2'-oxo-2H-spiro[furan-3,3'-indoline]-2-carboxylate

Trans(A5): Colorless solid. 44.0 % yield. ^{1}H NMR (500 MHz, DMSO-*d*$_6$) δ 7.83 (s, 1H), 7.10 – 6.99 (m, 1H), 6.97 (d, *J* = 7.8 Hz, 1H), 5.30 (s, 1H), 5.13 – 5.07 (m, 1H), 4.45 – 4.20 (m, 1H), 3.66 (q, *J* = 7.1 Hz, 1H), 1.80 (s, 1H), 1.67 (d, *J* = 1.6 Hz, 1H), 0.56 (t, *J* = 7.1 Hz, 1H). ^{13}C NMR (126 MHz, DMSO) δ 175.26, 168.70, 165.80, 142.46, 137.18, 130.09, 128.52, 124.33, 123.14, 118.61, 117.20, 109.73, 84.40, 61.24, 59.58, 54.39, 38.37, 25.80, 18.47, 13.39.

Cis(A6): Colorless solid. 31.9 % yield. ^{1}H NMR (500 MHz, DMSO-*d*6) δ 7.76 (s, 1H), 7.47 (dd, *J* = 7.5, 1.2 Hz, 1H), 7.34 (td, *J* = 7.8, 1.3 Hz, 1H), 6.94 (d, *J* = 7.8 Hz, 1H), 5.53 (s, 1H), 5.12 – 5.05 (m, 1H), 4.24 (qd, *J* = 15.4, 6.7 Hz, 1H), 3.99 (qt, *J* = 7.1, 3.7 Hz, 1H), 1.82 – 1.76 (m, 2H), 1.67 (d, *J* = 1.6 Hz, 2H), 1.03 (t, *J* = 7.1 Hz, 1H). 13C NMR (126 MHz, DMSO) δ 175.53, 168.78, 166.65,

143.06, 136.87, 130.10, 129.76, 124.54, 123.30, 118.71, 117.33, 109.48, 84.08, 61.59, 59.37, 55.03, 38.04, 25.86, 18.43, 14.07.

Trans(A5):

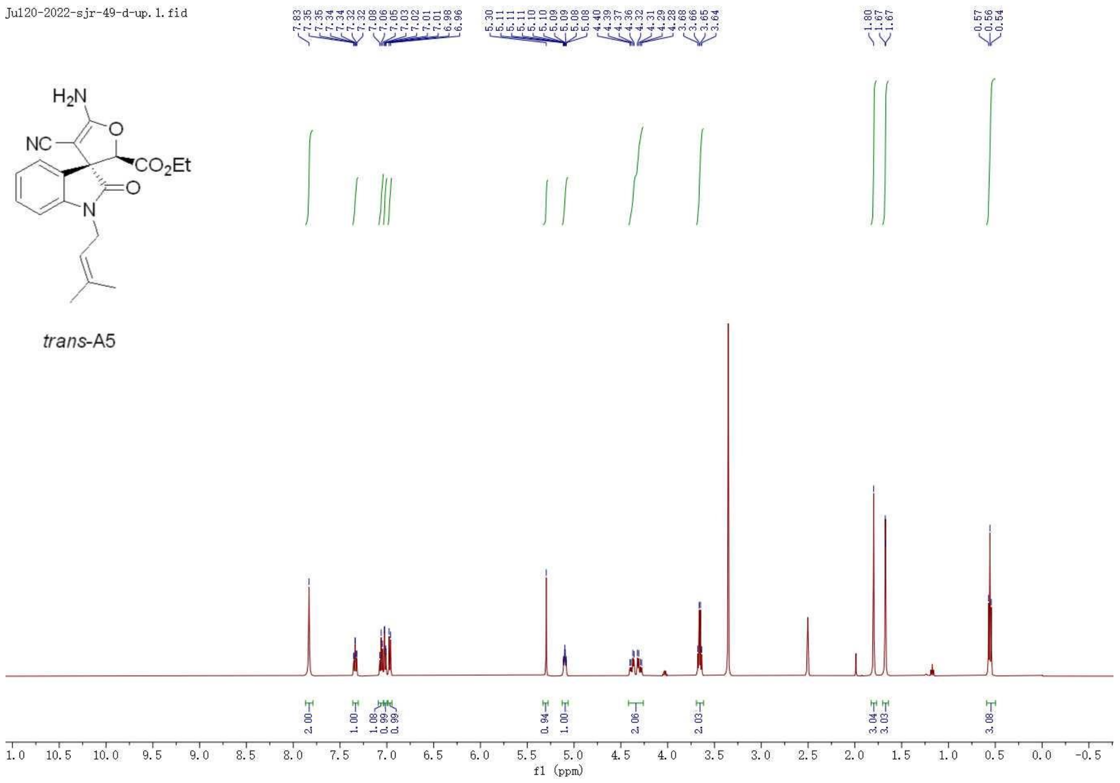

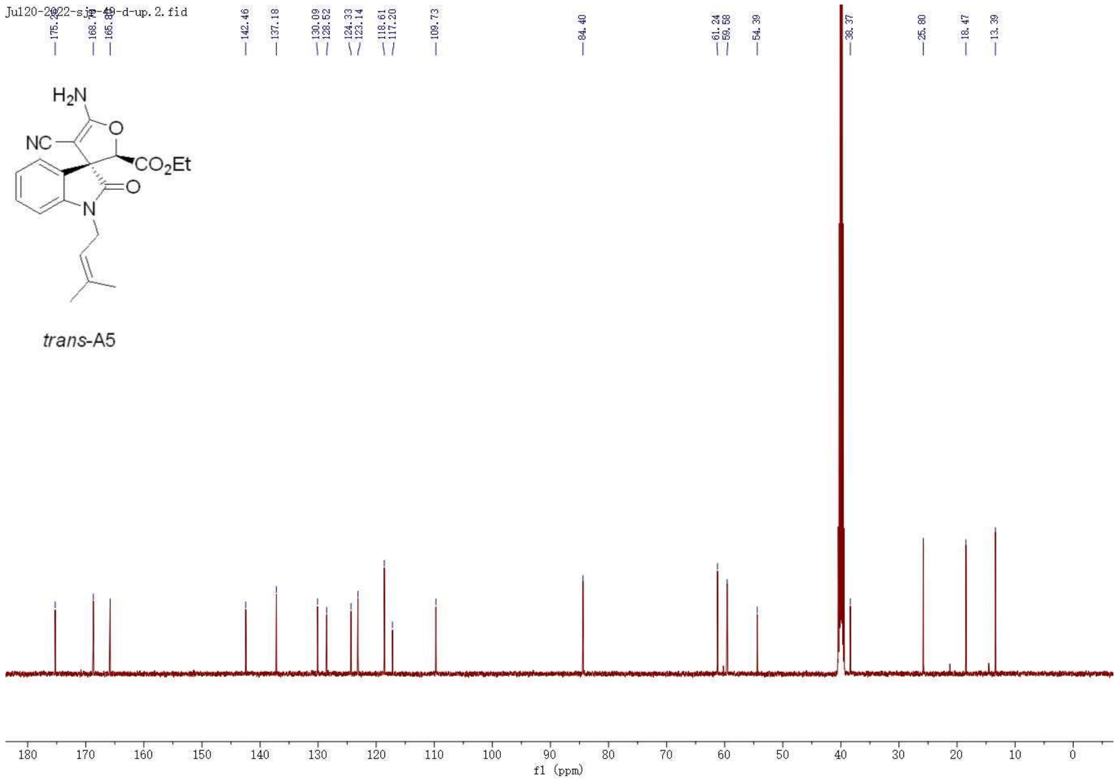

Cis(A6):

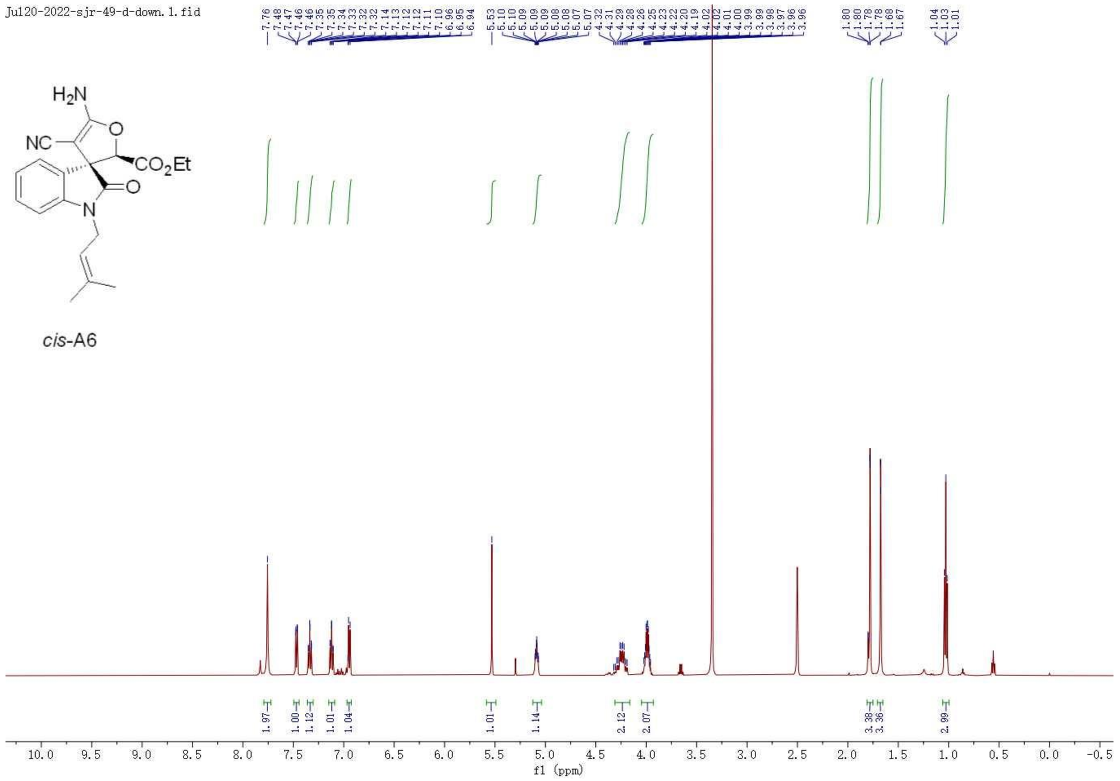
Jul20-2022-sjr-49-d-down.1.fid
cis-A6
f1 (ppm)

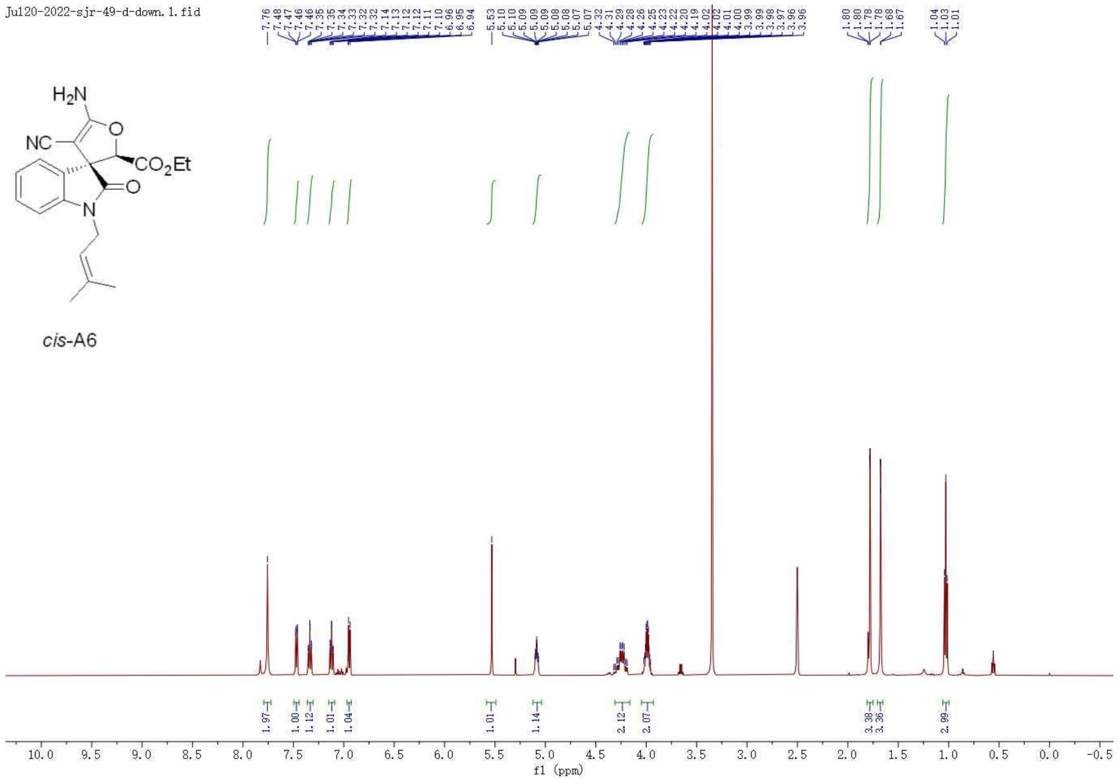
Jul20-2022-sjr-49-d-down.1.fid
cis-A6
f1 (ppm)

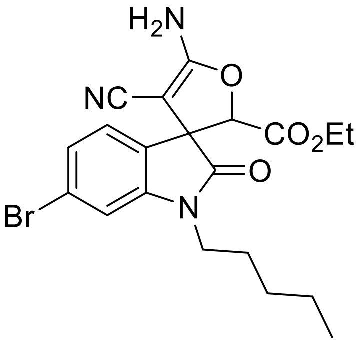

Column chromatography afforded the desired product A7+A8 in 76% yield as colorless solid, cis(A7) : trans(A8) = 42 :58

ethyl-5-amino-6'-bromo-4-cyano-2'-oxo-1'-pentyl-2H-spiro[furan-3,3'-indoline]-2-carboxylate

Trans(A7): Colorless solid. 44.0 % yield.^{1}H NMR (500 MHz, DMSO-d_6) δ 7.80 (s, 1H), 7.45 (d, J = 7.9 Hz, 1H), 7.38 (d, J = 1.8 Hz, 1H), 7.31 (dd, J = 7.9, 1.7 Hz, 1H), 5.57 (s, 1H), 4.11 – 3.93 (m, 1H), 3.65 (ddt, J = 46.2, 13.6, 7.0 Hz, 1H), 1.56 – 1.50 (m, 1H), 1.36 – 1.21 (m, 2H), 1.04 (t, J = 7.1 Hz, 1H), 0.84 (t, J = 6.8 Hz, 2H).^{13}C NMR (126 MHz, DMSO) δ 176.11, 168.74, 166.61, 144.95, 129.20, 126.49, 125.83, 122.73, 117.12, 112.44, 83.67, 61.66, 59.11, 54.81, 28.48, 26.93, 22.27, 14.27, 14.16.

Cis(A8): Colorless solid. 31.9 % yield. ^{1}H NMR (500 MHz, DMSO-d_6) δ 7.88 (s, 1H), 7.43 (d, J = 1.8 Hz, 1H), 7.27 (dd, J = 7.9, 1.7 Hz, 1H), 6.96 (d, J = 7.9 Hz, 1H), 5.30 (s, 1H), 3.94 – 3.60 (m, 2H), 1.56 (ddd, J = 11.7, 7.5, 3.7 Hz, 1H), 1.34 – 1.23 (m, 2H), 0.85 (t, J = 6.9 Hz, 2H), 0.64 (t, J = 7.1 Hz, 1H). ^{13}C NMR (126 MHz, DMSO) δ 175.77, 168.68, 165.77, 144.28, 127.78, 126.08, 125.79, 123.05, 116.97, 112.74, 84.04, 61.42, 59.30, 54.11, 28.45, 26.94, 22.27, 14.30, 13.54.

Trans(A7):

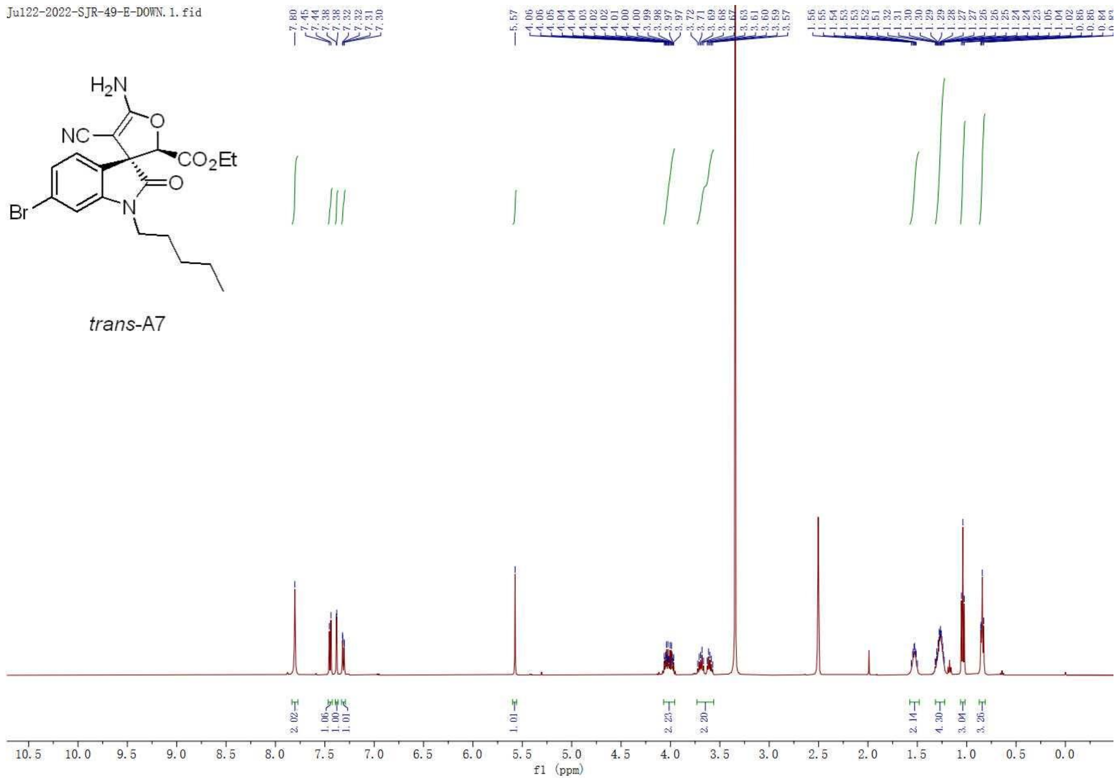

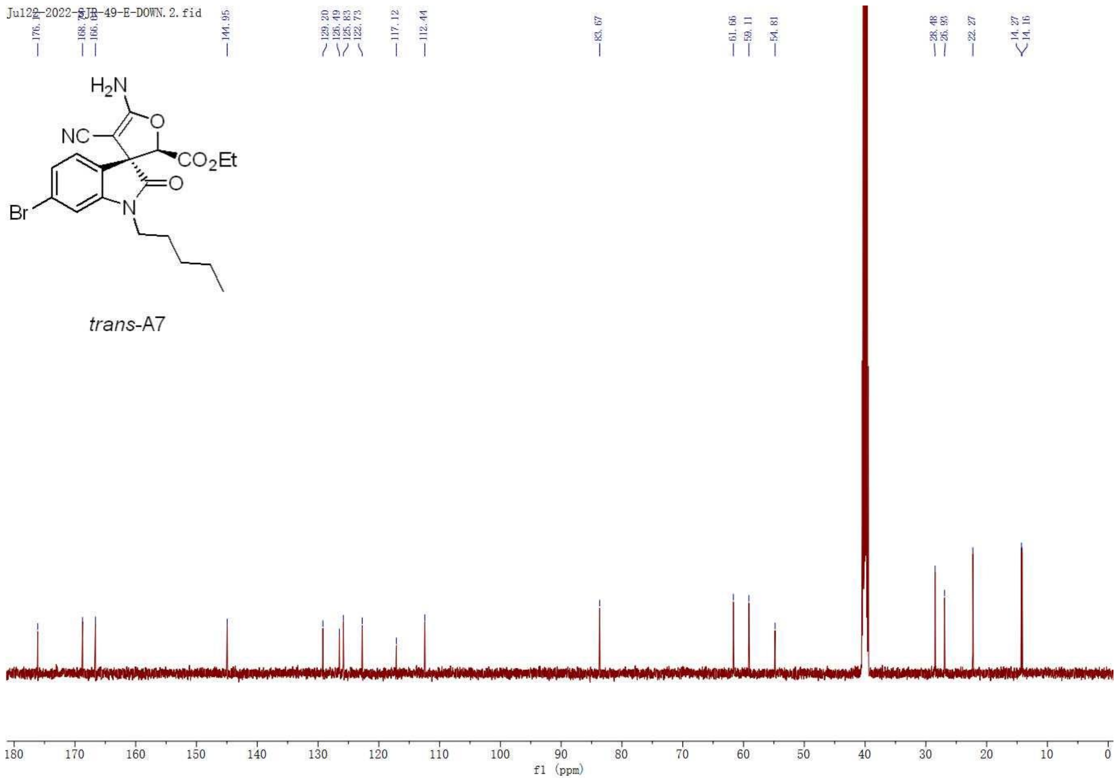

Cis(A8):

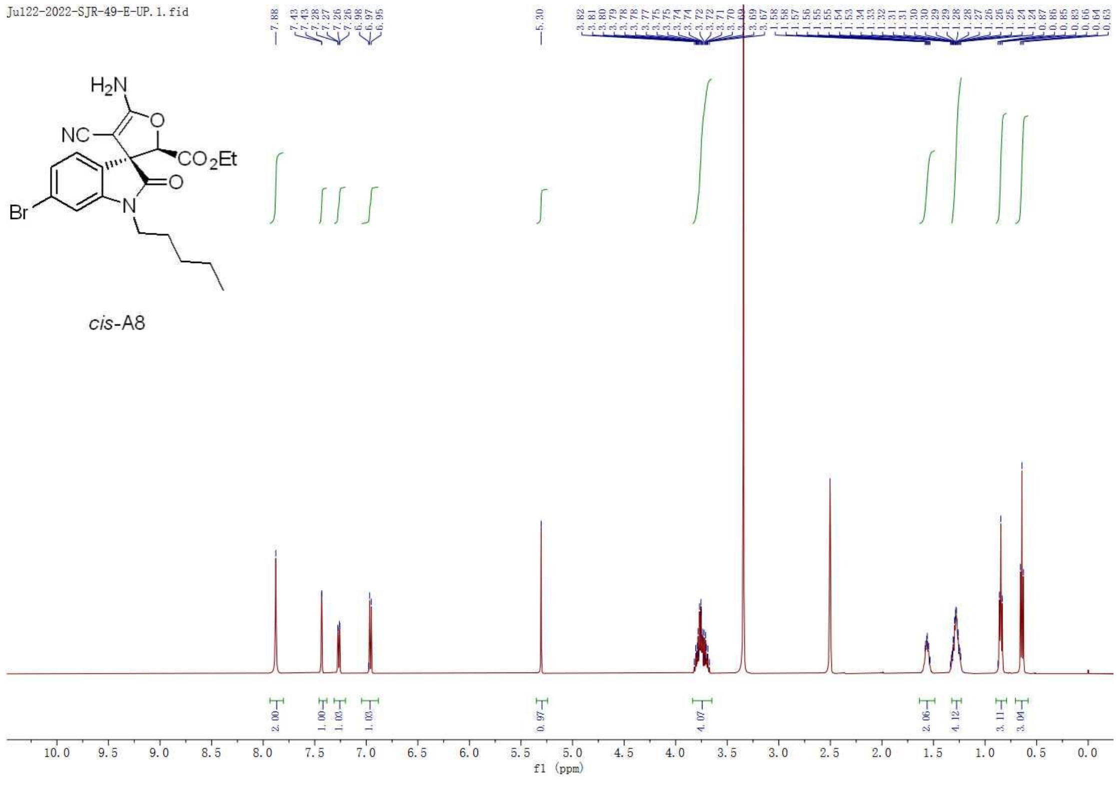

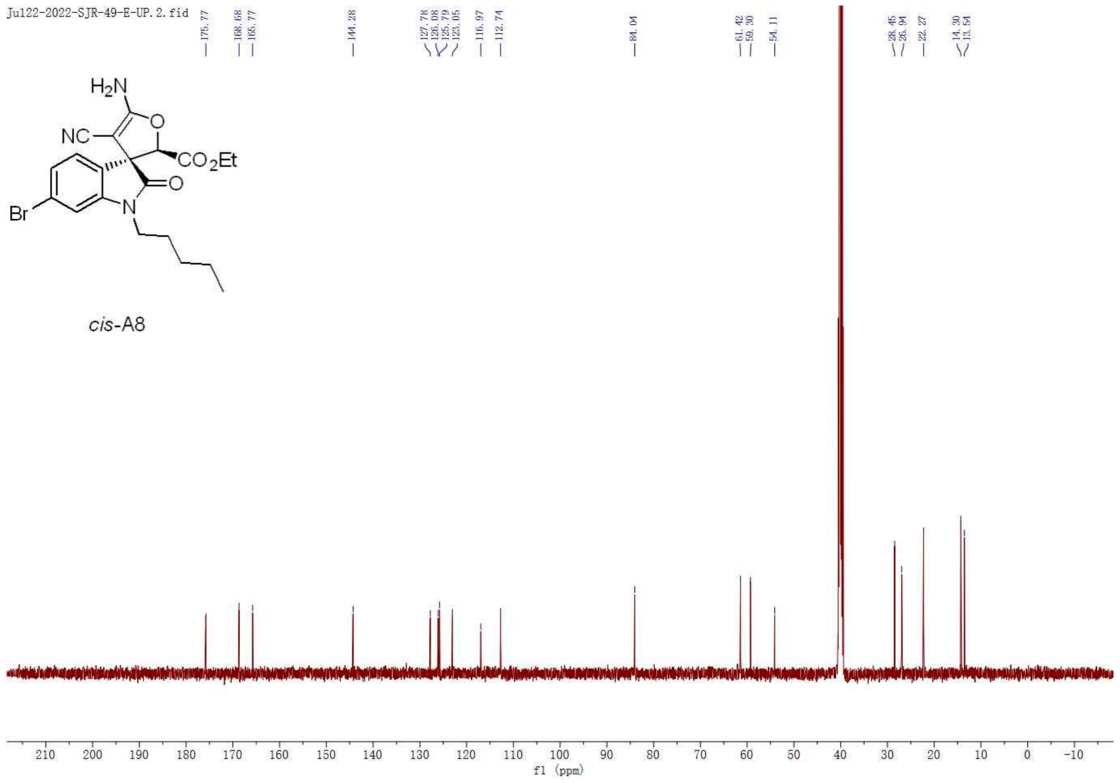

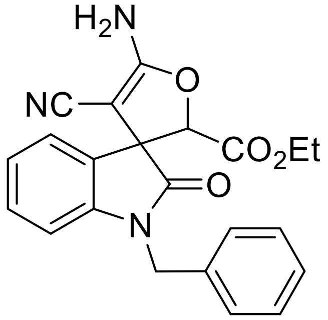

Column chromatography afforded the desired product A9+A10 in 85% yield as colorless solid, cis(A10) : trans(A9) = 52 : 48

Trans(A9): Colorless solid. 40.8 % yield. ^{1}H NMR (500 MHz, DMSO-d_6) δ 7.87 (s, 1H), 7.33 (d, *J* = 6.5 Hz, 2H), 7.08 – 7.02 (m, 1H), 6.93 (d, *J* = 7.9 Hz, 1H), 5.40 (s, 1H), 5.11 – 4.90 (m, 1H), 3.65 (ddq, *J* = 48.3, 10.8, 7.1 Hz, 1H), 0.38 (t, *J* = 7.1 Hz, 1H). ^{13}C NMR (101 MHz, DMSO) δ 176.10, 168.70, 165.83, 142.29, 136.22, 130.06, 129.02, 128.48, 127.98, 127.57, 124.42, 123.40, 117.26, 110.03, 84.31, 61.27, 59.67, 54.60, 43.60, 13.28.

Cis(**A10**): Colorless solid. 44.2 % yield. ^{1}H NMR (400 MHz, DMSO-d_6) δ 7.79 (s, 2H), 7.50 (dd, *J* = 7.5, 1.2 Hz, 1H), 7.37 – 7.29 (m, 4H), 7.26 (ddt, *J* = 7.7, 6.4, 1.6 Hz, 2H), 7.11 (td, *J* = 7.5, 1.0 Hz, 1H), 6.88 (d, *J* = 7.7 Hz, 1H), 5.61 (s, 1H), 5.00 (d, *J* = 15.9 Hz, 1H), 4.80 (d, *J* = 15.9 Hz, 1H), 4.00 (qd, *J* = 7.1, 2.8 Hz, 2H), 1.01 (t, *J* = 7.1 Hz, 3H). 13C NMR (101 MHz, DMSO-d6) δ 176.32, 168.78, 166.76, 142.93, 136.27, 130.05, 129.73, 129.01, 127.87, 127.51, 124.66, 123.54, 117.41, 109.82, 84.04, 61.66, 59.56, 55.26, 43.29, 14.15.

Trans(A9):

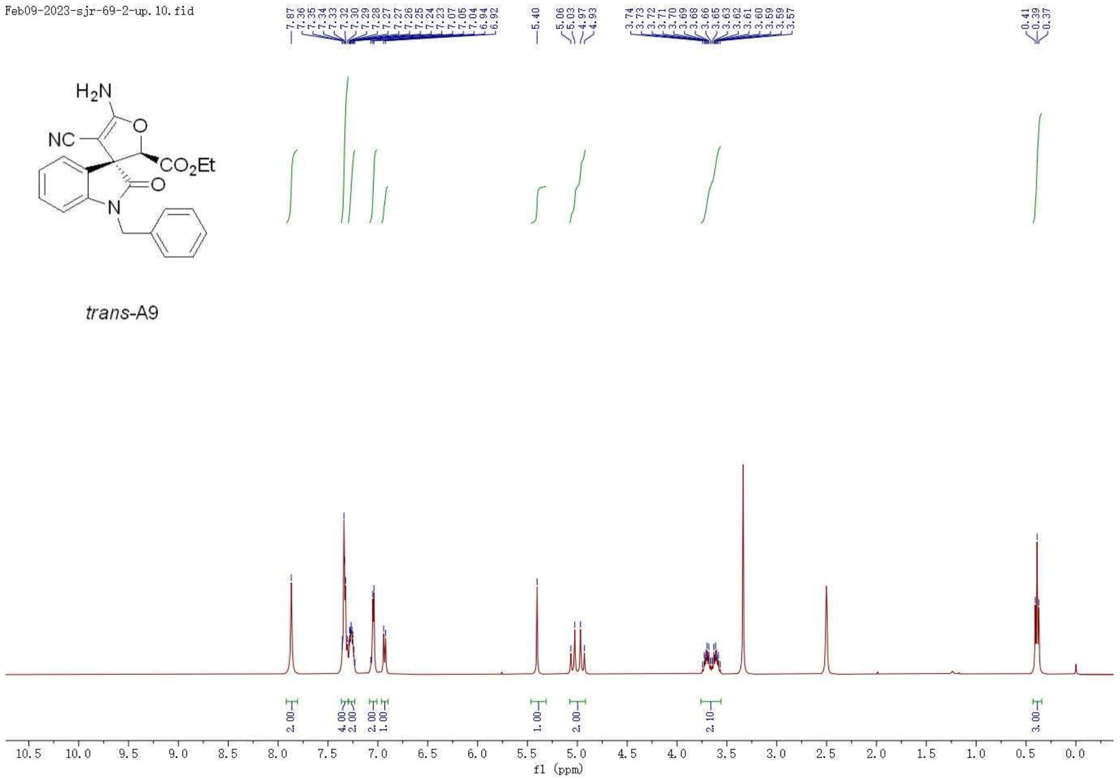

Feb09-2023-sjr-69-2-up.10.fid
7.87 7.36 7.35 7.34 7.33 7.32 7.30 7.29 7.28 7.27 7.27 7.26 7.25 7.24 7.23 7.07 7.05 7.04 6.94 6.92
5.40
5.06 5.03 4.97 4.93
3.74 3.73 3.72 3.71 3.70 3.69 3.68 3.66 3.65 3.63 3.62 3.61 3.60 3.59 3.59 3.57
0.41 0.39 0.37
H_2N
NC
O
CO_2Et
O
N
trans-A9
2.00 4.00 2.00 2.00 1.00 1.00 2.00 2.10 3.00
10.5 10.0 9.5 9.0 8.5 8.0 7.5 7.0 6.5 6.0 5.5 5.0 4.5 4.0 3.5 3.0 2.5 2.0 1.5 1.0 0.5 0.0
f1 (ppm)

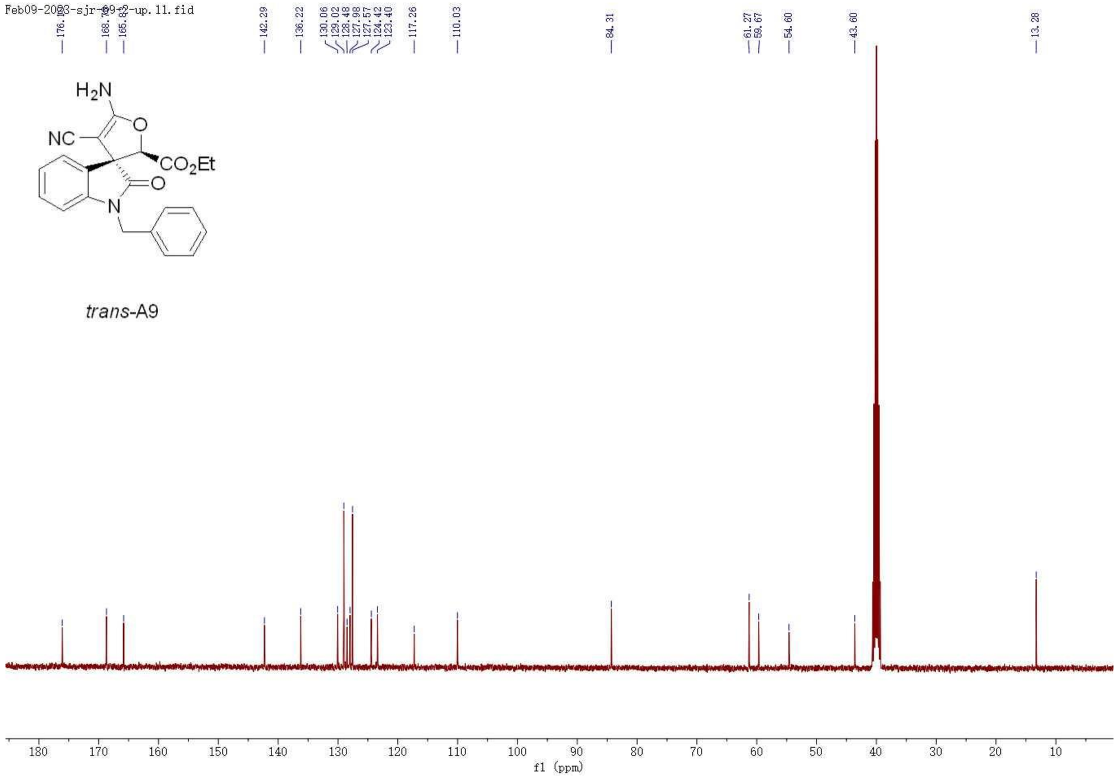

Feb09-2023-sjr-69-2-up.11.fid
168.70 165.82
142.29
136.22
130.06 129.02 128.48 127.98 127.57 124.42 123.40
117.26
110.03
84.31
61.27 59.67
54.60
43.60
13.28
H_2N
NC
O
CO_2Et
O
N
trans-A9
180 170 160 150 140 130 120 110 100 90 80 70 60 50 40 30 20 10
f1 (ppm)

Cis(A10):

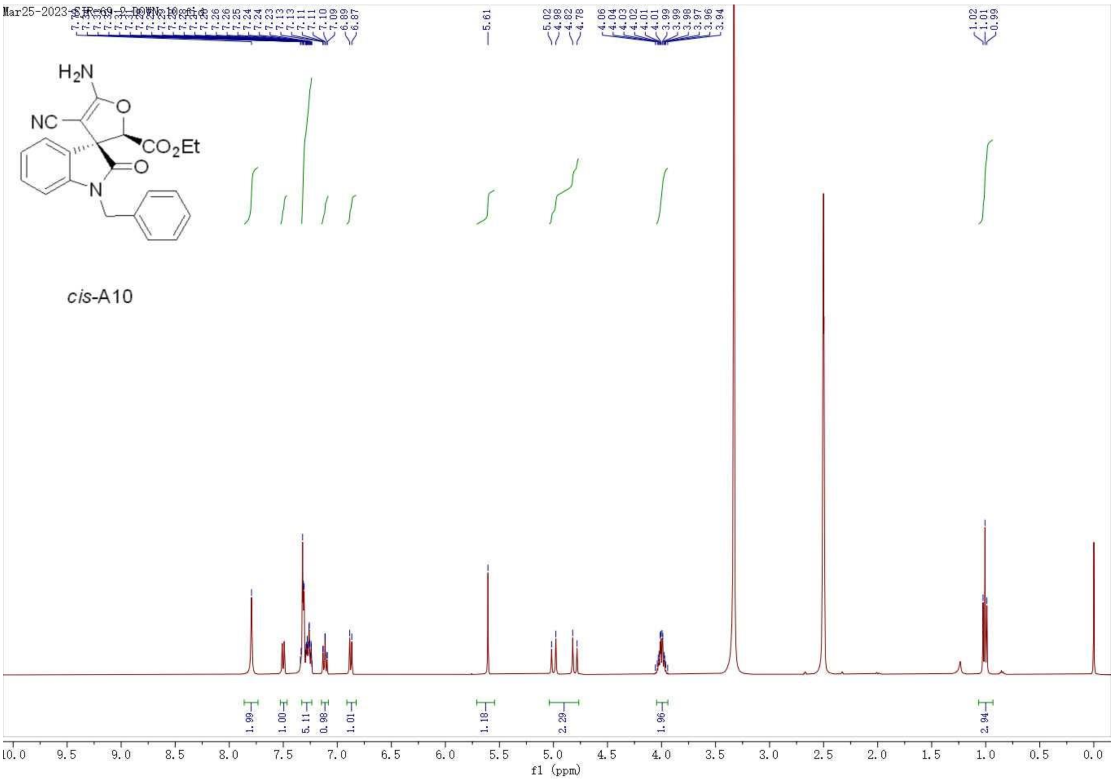
Mar25-2023-SJR-69-2-DOWN.10.fid
H_2N
NC
CO_2Et
O
N
cis-A10
f1 (ppm)

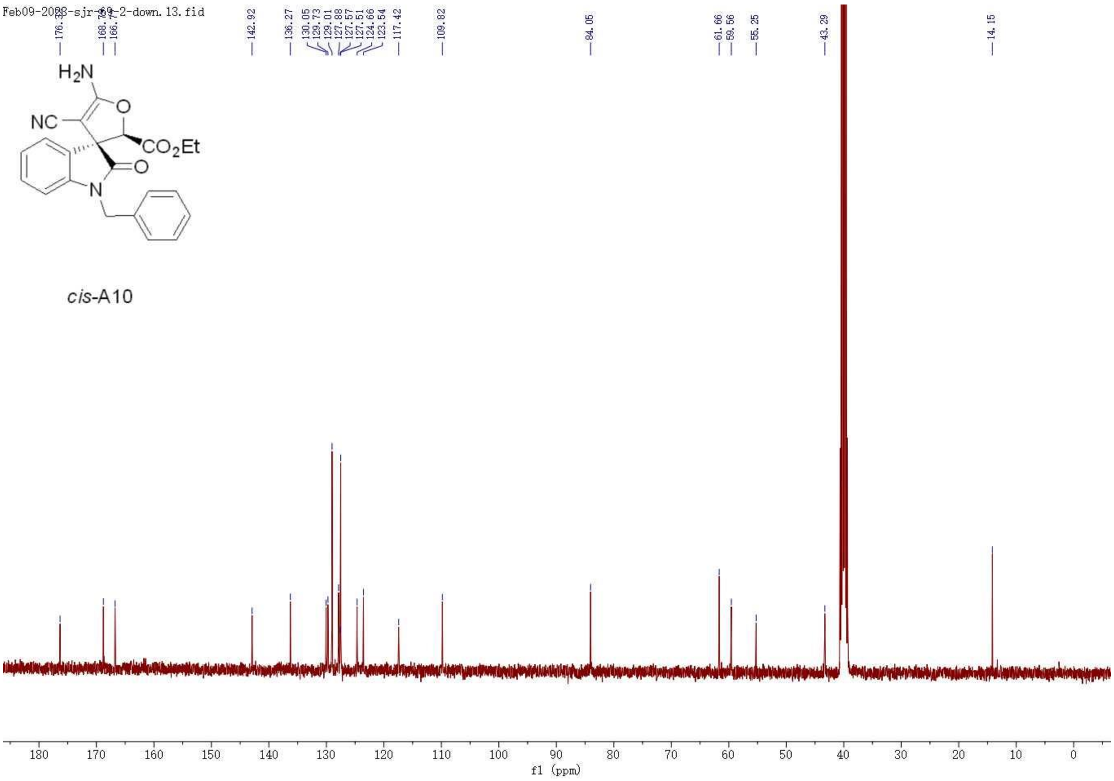
Feb09-2023-sjr-69-2-down.13.fid
142.92
136.27
130.05
129.73
129.01
127.88
127.57
127.51
124.66
123.54
117.42
109.82
84.05
61.66
59.56
55.25
43.29
14.15
H_2N
NC
CO_2Et
O
N
cis-A10
f1 (ppm)

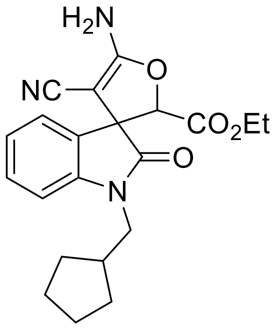

A14

ethyl 5-amino-4-cyano-1'-(cyclopentylmethyl)-2'-oxo-2H-spiro[furan-3,3'-indoline]-2-carboxylate

Column chromatography afforded the desired product A14+A15 in 83% yield as colorless solid, cis(A15) : trans(A14) = 46 : 54

Trans(A14): Colorless solid. 44.8 % yield. 1H NMR (500 MHz, DMSO-d6) δ 7.73 (s, 1H), 7.47 (d, J = 7.4 Hz, 1H), 7.33 (t, J = 7.8 Hz, 1H), 7.11 (t, J = 8.0 Hz, 1H), 5.53 (s, 1H), 4.00 (ddt, J = 39.0, 10.9, 6.7 Hz, 1H), 3.77 – 3.42 (m, 1H), 1.74 – 1.54 (m, 2H), 1.24 (s, 1H), 1.02 (t, J = 7.1 Hz, 2H). 13C NMR (101 MHz, DMSO) δ 176.23, 168.69, 166.75, 143.60, 130.04, 129.74, 124.52, 123.17, 117.30, 109.46, 84.00, 61.49, 59.44, 55.33, 44.34, 38.25, 30.13, 30.04, 24.86, 24.83, 14.15.

Cis(**A15**): Colorless solid. 38.2 % yield. ^{1}H NMR (500 MHz, DMSO-d_6) δ 7.80 (s, 1H), 7.34 (t, J = 7.7 Hz, 1H), 7.14 (d, J = 7.9 Hz, 1H), 7.04 (dd, J = 17.7, 7.4 Hz, 1H), 5.29 (s, 1H), 3.69 (dd, J = 13.2, 7.1 Hz, 2H), 2.31 (p, J = 7.6 Hz, 1H), 1.66 – 1.62 (m, 2H), 1.50 – 1.47 (m, 1H), 1.29 – 1.26 (m, 1H), 0.56 (t, J = 7.1 Hz, 1H). ^{13}C NMR (126 MHz, DMSO) δ 176.01, 168.64, 165.92, 142.91, 130.10, 128.41, 124.35, 123.04, 117.19, 109.73, 84.31, 61.26, 59.57, 54.64, 44.70, 38.27, 30.07, 30.02, 24.82.

Trans(A14):

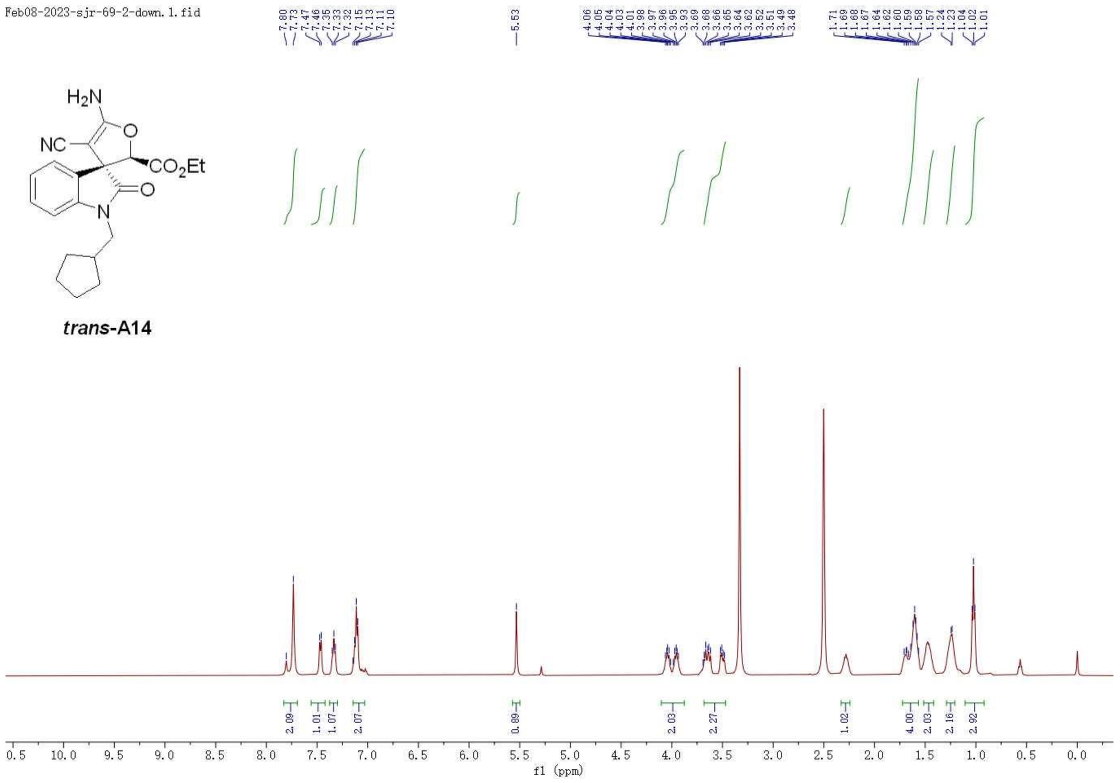

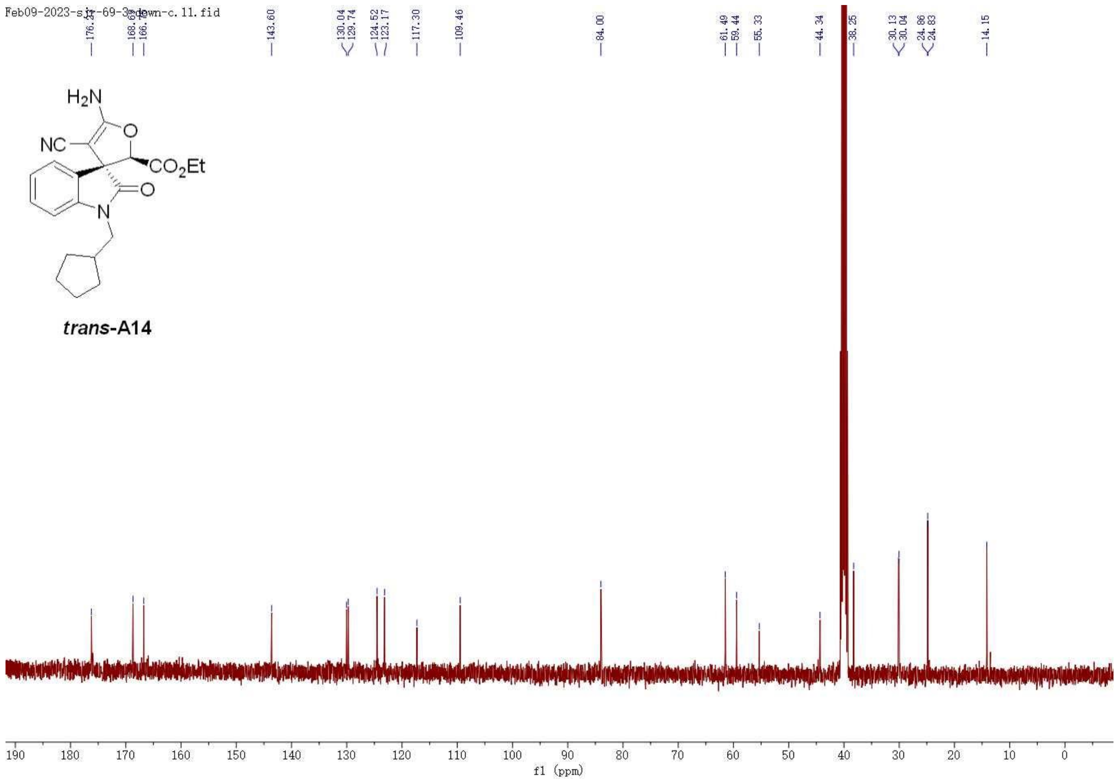
Feb09-2023-sjr-69-3-down-c.11.fid
143.60
130.04
129.74
124.52
123.17
117.30
109.46
84.00
61.49
59.44
55.33
44.34
38.25
30.13
30.04
24.86
24.83
14.15
H2N
NC
O
CO2Et
O
N
trans-A14
f1 (ppm)

Cis(A15):

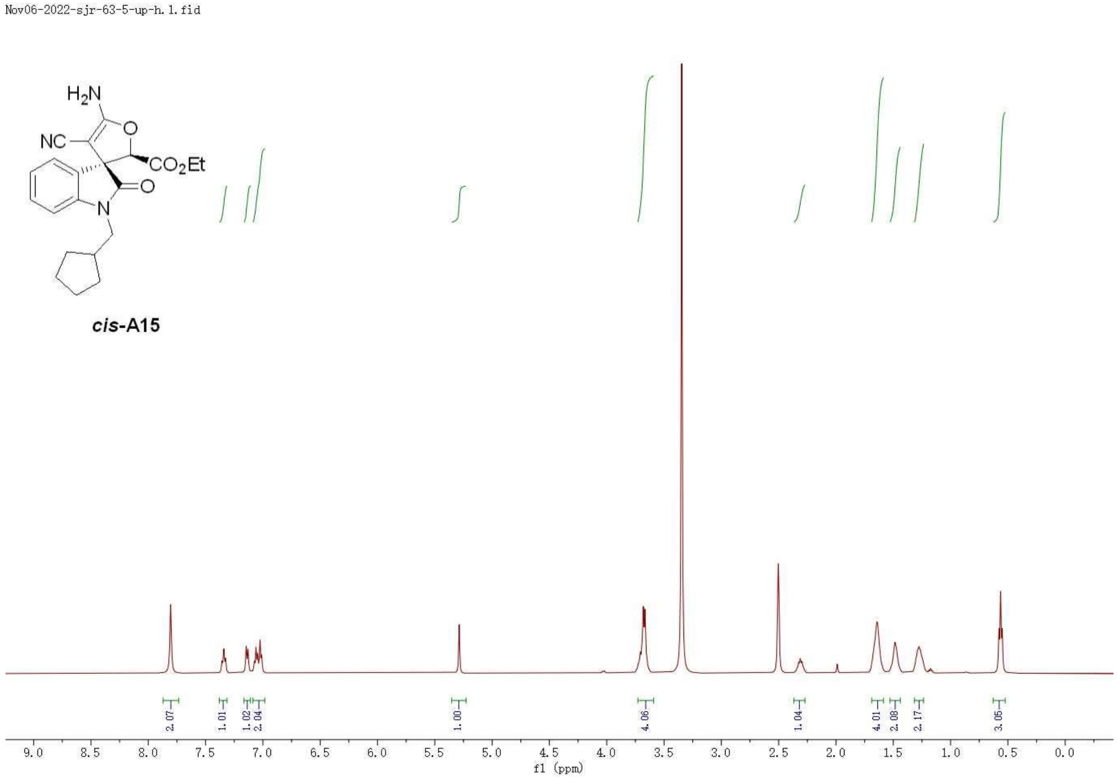
Nov06-2022-sjr-63-5-up-h.1.fid
H2N
NC
O
CO2Et
O
N
cis-A15
2.07
1.01
1.02
2.04
1.00
4.06
1.04
4.01
2.08
2.17
3.05
f1 (ppm)

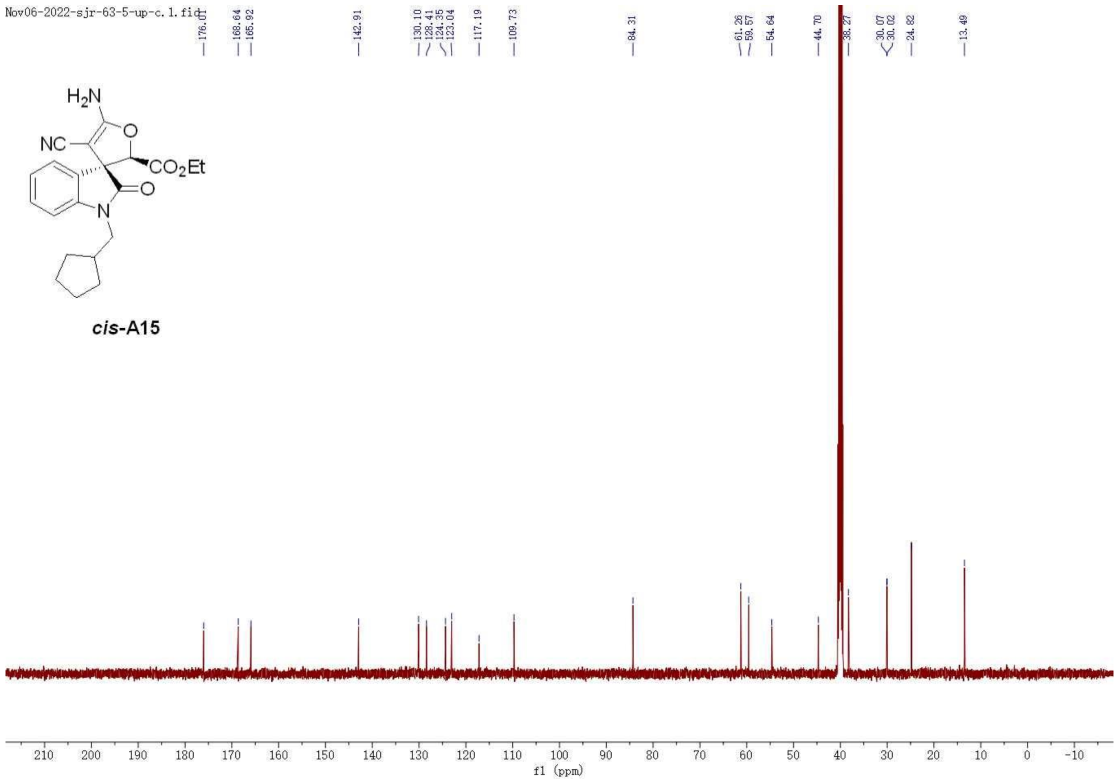

Previous reported compounds

A1,A2,A11,A12,A13,A16,A17 was confirmed to be consistent with compound **trans-6a, cis-6a, , trans-6e, cis-6e, trans-6k, cis-6k, trans-6o** in origin work by 1H-NMR spectrum[8].

B1-B6 was confirmed to be consistent with compound **3g, 3p, 3c, 5, 7, 8**in origin work by 1H-NMR spectrum[21].

C1-C16 was confirmed to be consistent with compound **4ua, 4ta, 4wa, 4sa, 4aa, 7a, 4oa, 4af, 4am, 4ra, 4ka, 4pa, 4ao, 13, 12, 4qa** in origin work by 1H-NMR spectrum[2].

D1-D5 was confirmed to be consistent with compound **7c, 7e, 6e, 5b, 5k** in origin work by 1H-NMR spectrum[22].

Representative compounds

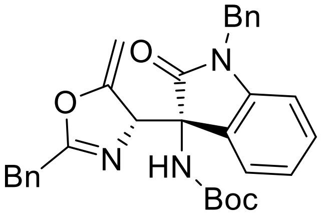

tert-Butyl ((R)-1-benzyl-3-((S)-2-benzyl-5-methylene-4,5-dihydrooxazol-4-yl)-2-oxoindolin-3-yl)carbamate (**C-4**) White solid, 62% yield, >99% ee ; 1H NMR (400 MHz, CDCl3) (δ, ppm) 7.39 (d, J = 6.8 Hz, 2H), 7.31 – 7.13 (comp, 8H), 7.09 (d, J = 6.2 Hz, 2H), 7.02 – 6.92 (m, 1H), 6.64 (d, J = 7.8 Hz, 1H), 5.90 (s, 1H), 5.12 (d, J = 15.6 Hz, 1H), 4.98 – 4.87 (m, 1H), 4.66 (d, J = 15.0 Hz, 1H), 4.46 (t, J = 2.4 Hz, 1H), 3.68 – 3.54 (m, 3H), 1.26 (s, 9H).

^{13}C NMR (101 MHz, $CDCl_3$) δ 166.78, 155.90, 143.87, 135.77, 133.67, 129.36, 129.04, 128.58, 128.47, 127.68, 127.44, 127.07, 122.83, 122.71, 109.04, 88.35, 80.57, 71.84, 62.88, 44.20, 34.59, 28.05.**C-4** was confirmed to be consistent with compound **4sa** in origin work confirmed by 1H-NMR spectrum and ^{13}C-NMR spectrum.

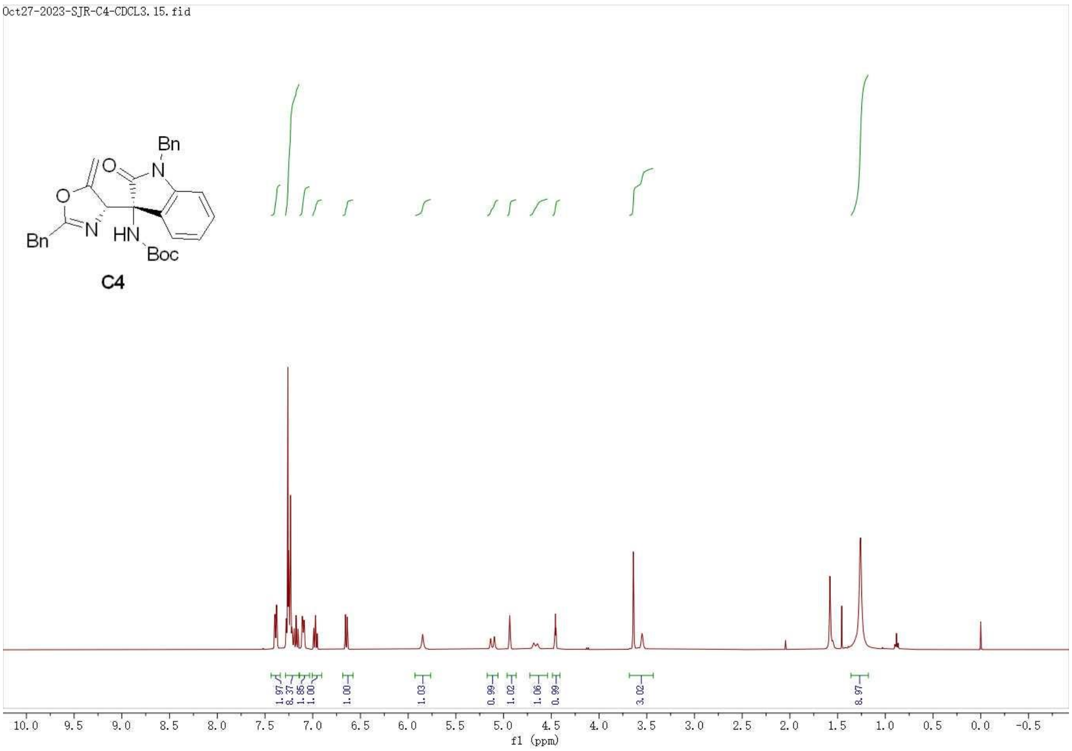

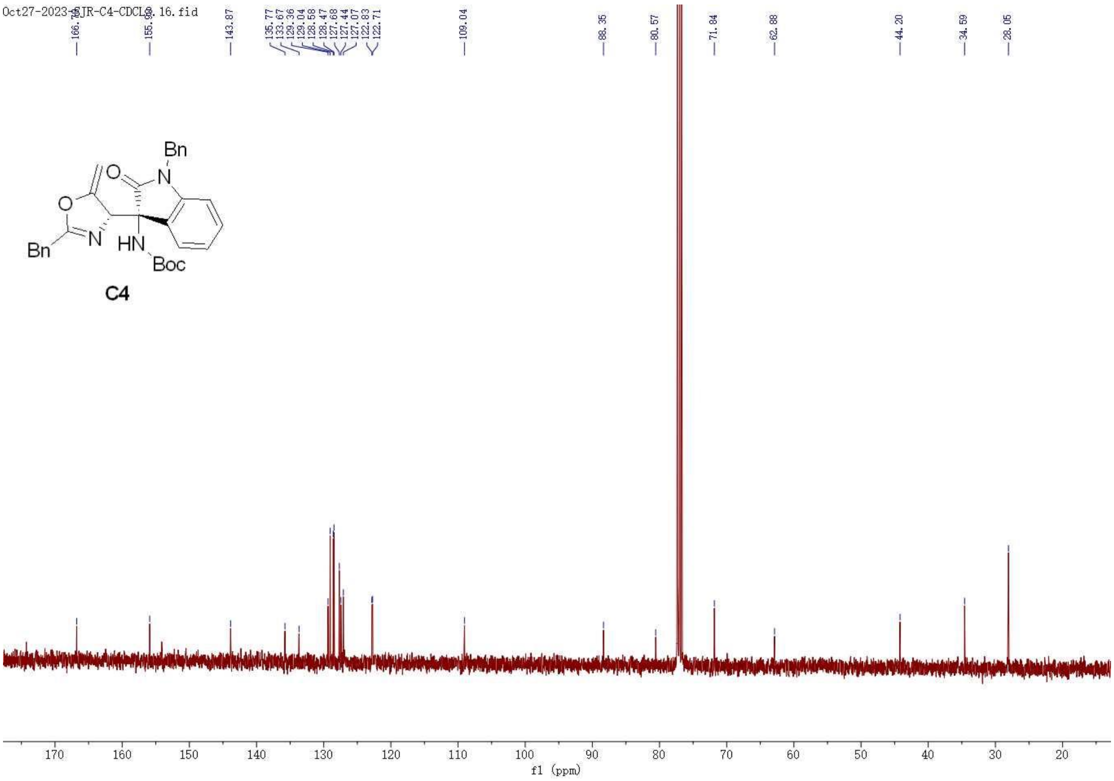

3. General MD simulation results of C4-5EK0 complex

To further verify the accuracy of the docking result, a MD simulation was taken. Root means square deviation (RMSD) represents the sum of all atomic deviations between the conformation at a certain moment and the target conformation and is an important basis for measuring the stability of the system. **5EK0-C4** was chosen to be the example. The RMSD of protein molecules in both systems over time is shown in Supplementary Figure 1 The RMSD of proteins in the **5EK0-C4** system showed significant fluctuations throughout the first 40 ns of the simulation, and the fluctuations decreased after 40 ns, gradually stabilizing. It is speculated that during the first 40 ns, the conformation of the solvent molecules and small molecules interacted with the protein, causing a certain change in their conformation, making the binding between the two more stable. The average RMSD values of the two systems between 60 and 100 ns is 1.141 ± 0.127 nm.

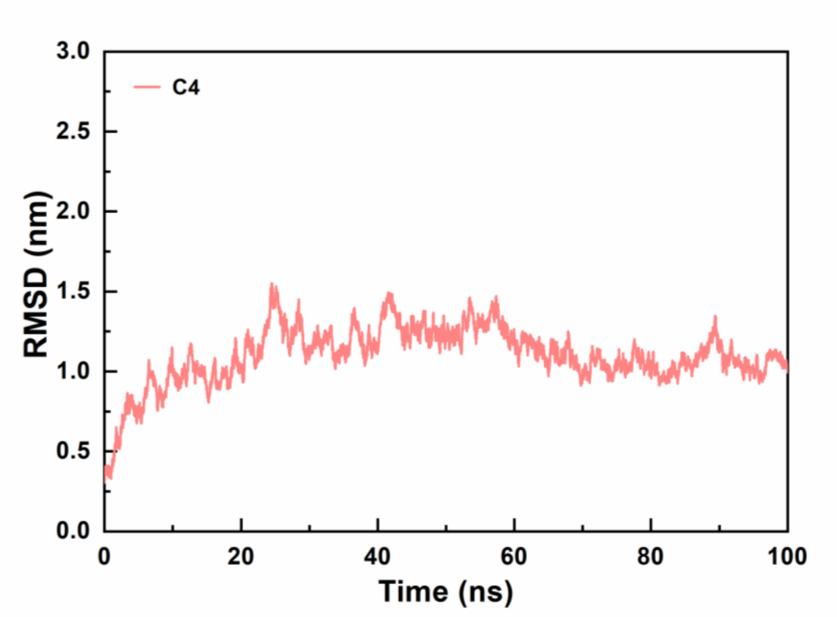

Supplementary Figure 1. Variations in the RMSD values of complex **5EK0-C4** over the course of the MD simulation.

Due to the unique structure of the protein and the docking method, these result in a high RMSD value of the ligand complex. To clarify the stability of the VSD4 binding domain, we calculated the RMSD of the ligand binding region (Met1493-Val1623, Supplementary Figure 2). The RMSD of the binding region in the **5EK0-C4** system fluctuated slightly throughout the first 40 ns of the

simulation. Within the initial 10 ns, the RMSD value increased to 0.4 nm, followed by an increase to 0.6 nm around 40 ns, and finally gradually stabilized. The overall RMSD change amplitude is relatively low compared to the whole protein, indicating that the change in the binding region of the **5EK0-C4** system is stable, with an average of 0.604 ± 0.022 after 60 ns.

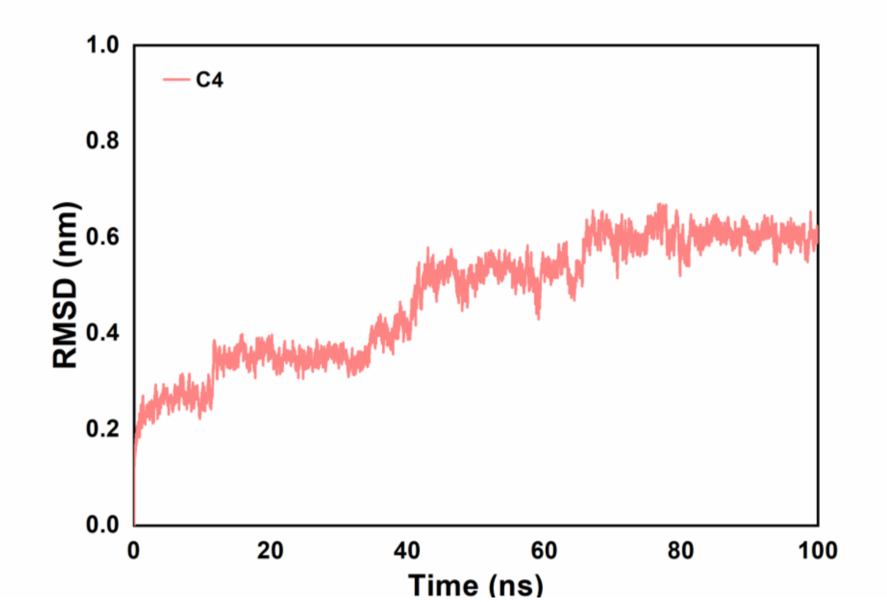

Supplementary Figure 2. Changes in RMSD values of the VSD4 binding domains (**5EK0-C4**) during MD simulation.

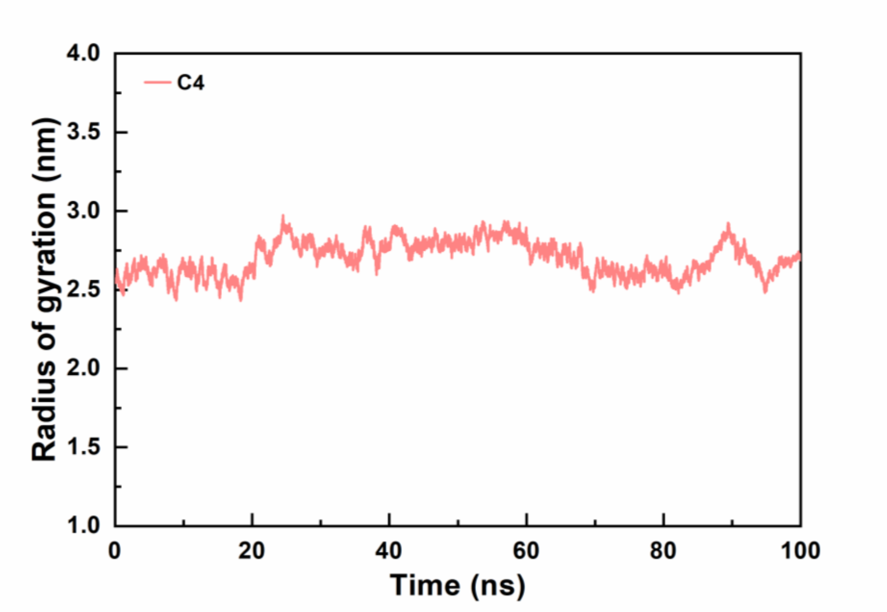

Supplementary Figure 3. Changes in Radius of gyration (Rg).

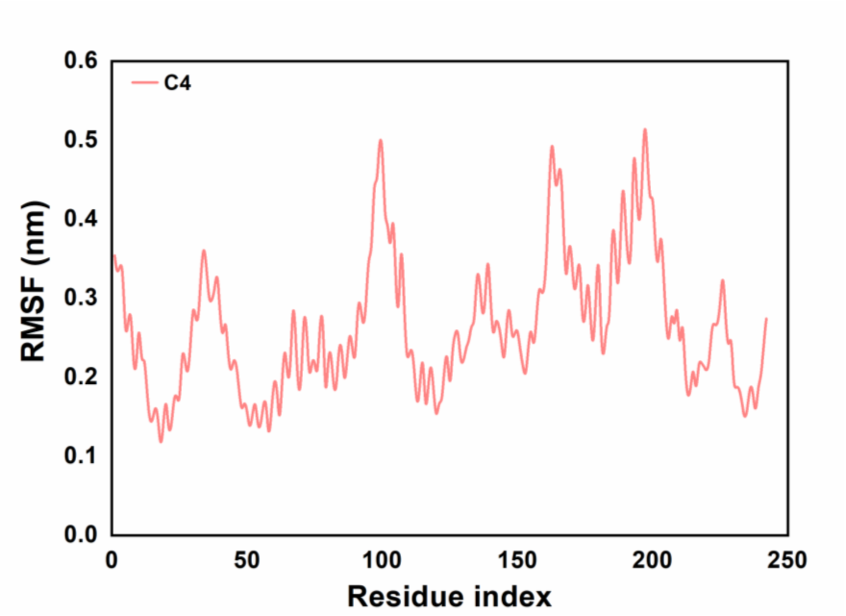

Supplementary Figure 4. RMSF values of all amino acid residues

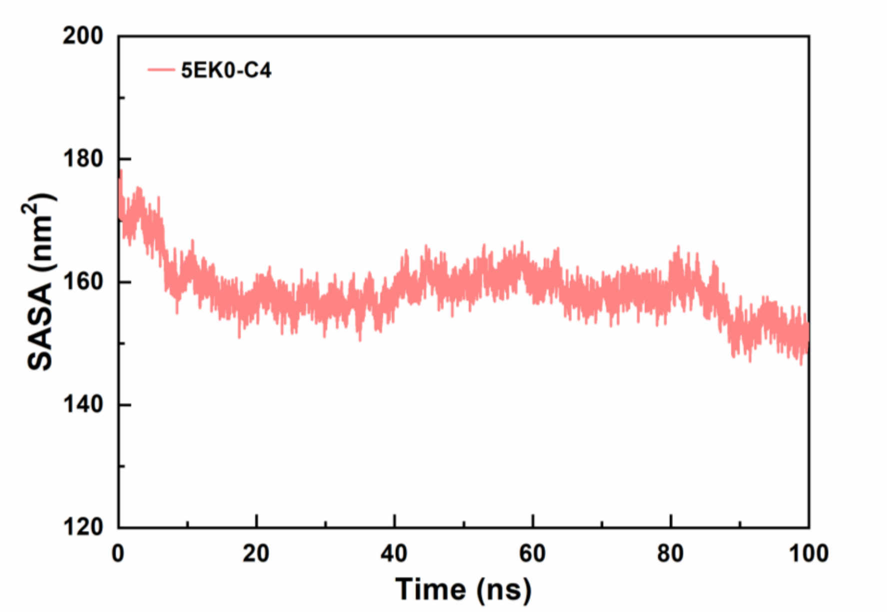

Supplementary Figure 5. Changes in SASA values of composite systems over simulation time

The overall calculation results indicate that the dynamic simulation based on this force field parameter is stable and reliable, and the whole system can be used for further analysis. Next, we calculated the binding free energy between proteins and small molecules at the 30-50 ns interval of RMSD in the two systems with the help of MM/PBSA method. The total binding free energy between small molecules and proteins in the **5EK0-C4** system is -129.204 kJ/mol, and the electrostatic interaction of the system includes the electrostatic interaction under vacuum conditions and polar Solvation energy (ΔGele+ ΔGPB) is 18.338 kJ/mol; Non polar interaction can be used as hydrophobic interaction energy, including Van der Waals force interaction and non-

polar Solvation free energy (ΔGvdw+ ΔGnp), the non-polar interaction is -147.542 kJ/mol. The values of each energy term can be found in Supplementary Table 2.

Supplementary Table 2 Various energy terms of binding free energy between small molecules and proteins

System	**5KE0-C4**	
Item	Energy(kJ/mol)	Delta
ΔG_{vdw}(kJ/mol)	-132.223	19.590
ΔG_{ele}(kJ/mol)	-129.364	18.242
ΔG_{PB}(kJ/mol)	147.702	19.257
ΔG_{np}(kJ/mol)	-15.319	0.658
**ΔG_{bind}(kJ/mol)**	**-129.204**	**7.458**

The binding free energy between proteins and small molecules was calculated using the MM/PBSA method in the stable range of 30-50 ns for RMSD in two systems.

4. PCA details information

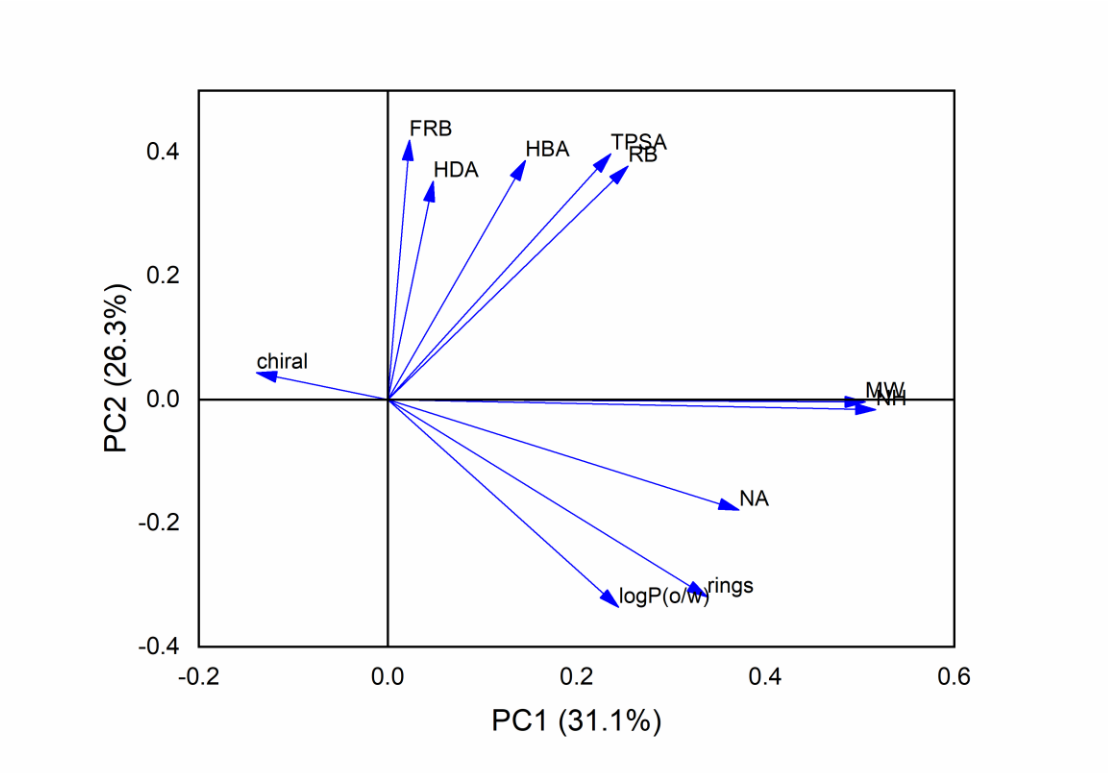

Supplementary Figure 6. Loading plot for the weight of 11 descriptors in PCA

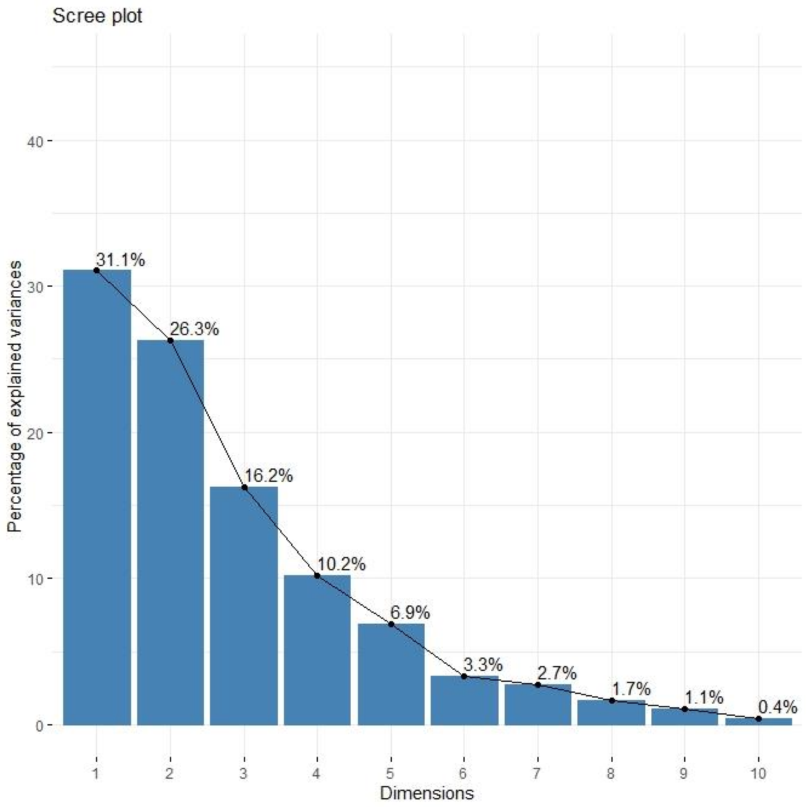

Supplementary Figure 7. Screeplot of PCA

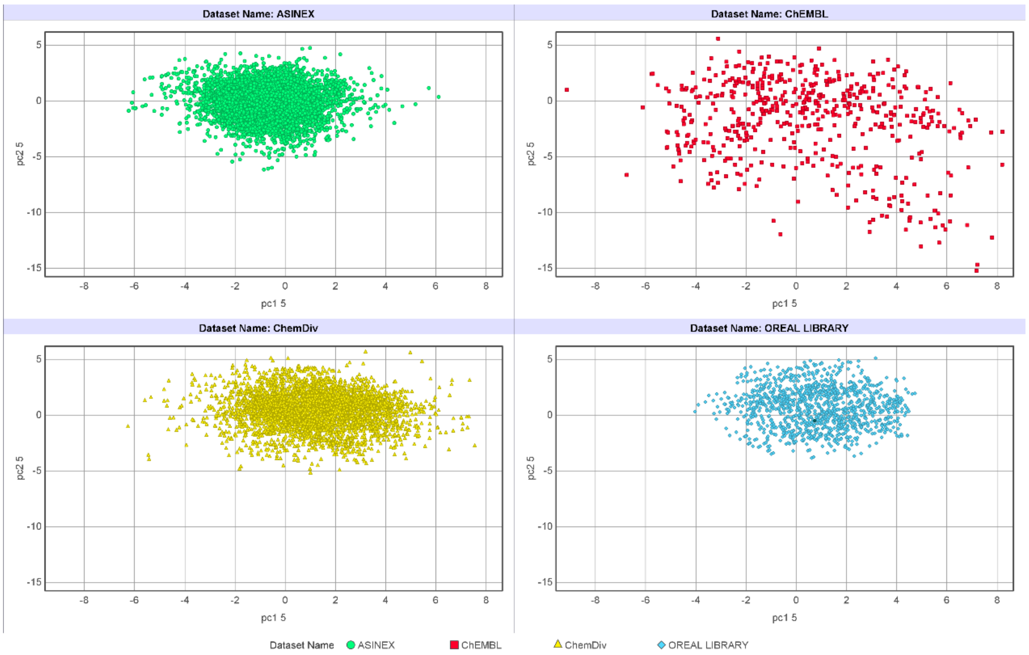

Supplementary Figure 8. PCA analysis results for each compound library

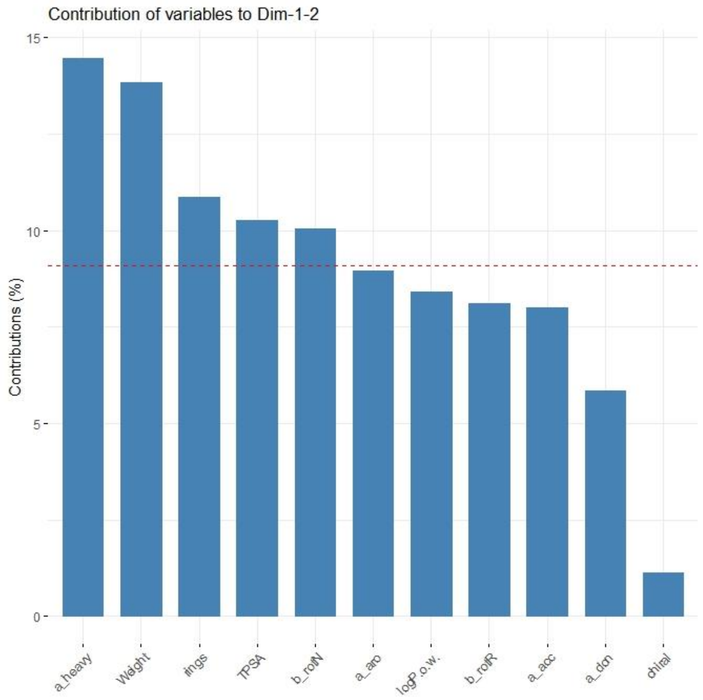

Supplementary Figure 9. The contribution of variables to principal components. The red dashed line represents the expected average contribution, and if the variable exceeds this line, it is considered to have an important contribution to the principal component11 descriptors were used for PCA, including hydrogen bond donors (a_don), hydrogen bond acceptors (a_acc), number of rotatable bonds (b_rotN), molecular weight (Weight), octanol/water partition coefficient (logP(o/w)), and topological polar surface area (TPSA), number of heavy atom (a_heavy), number of chiral center (chiral), fraction of rotatable bond (b_rotR), number of ring(ring) and number of aromatic atom (a_aro).

5. virtual screening result

Supplementary Table 3.42 virtual hits docking result and cell calcium imaging assays

Compound no.	Chemical structure	MW	Docking Score	Inhibition (%) VTD-evoke
A1	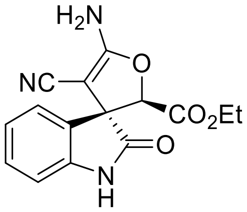	299.09	-7.0109	-10.039
A2	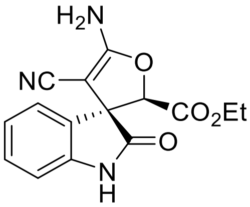	299.09	-7.0111	-7.033
A3	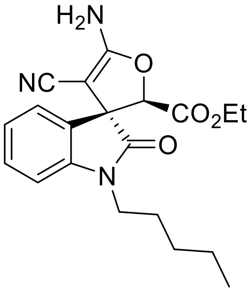	369.17	-7.6796	5.433
A4	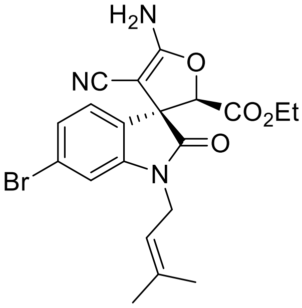	445.06	-7.1542	-19.794
A5	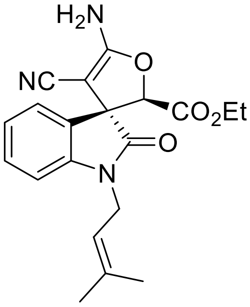	367.15	-8.5834	-63.175

A6	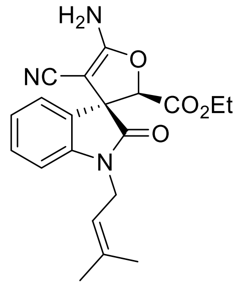	367.15	-8.3166	0.480
A7	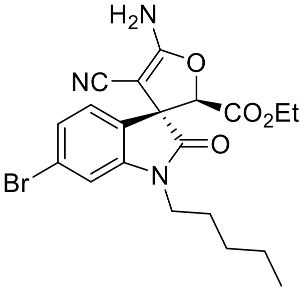	447.08	-7.0033	-1.469
A8	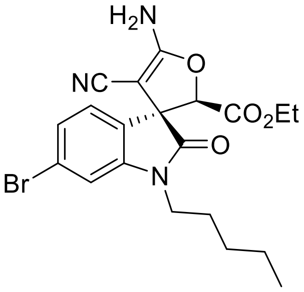	447.08	-6.9987	2.754
A9	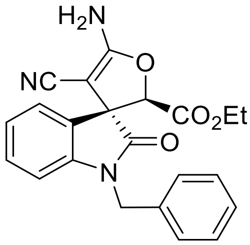	389.41	-7.7451	-16.960
A10	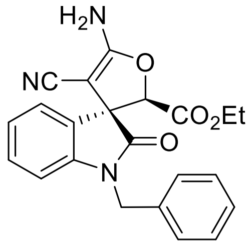	389.41	-8.7012	26.449
A11	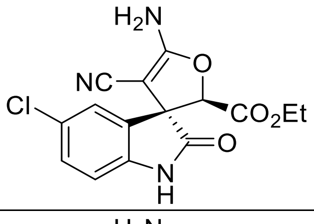	333.05	-7.0653	13.345
A12	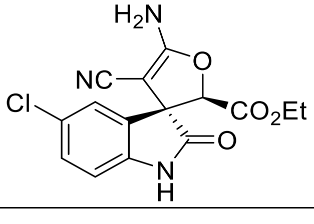	333.05	-7.1156	12.031

A13	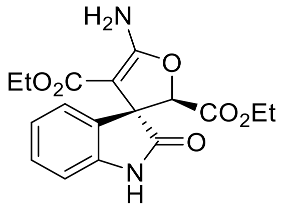	346.12	-7.2163	10.980
A14	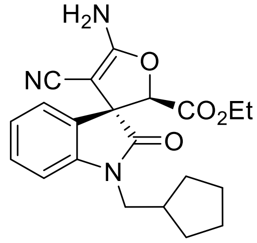	381.17	-7.8131	22.415
A15	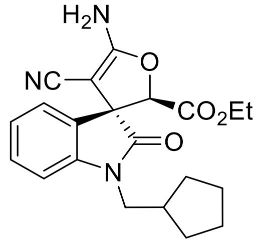	381.17	-7.7710	6.845
A16	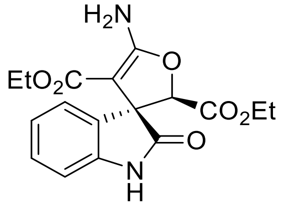	346.12	-7.2180	7.703
A17	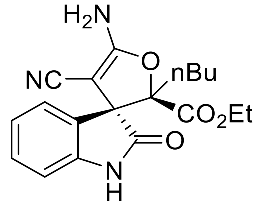	355.15	-7.2631	28.732
B1	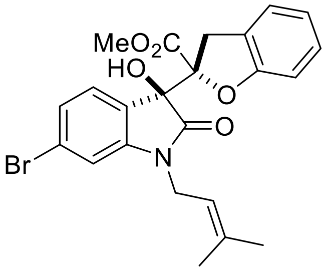	471.07	-8.2290	19.250
B2	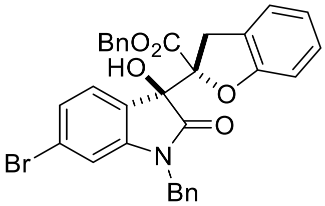	569.08	-7.1138	10.931
B3	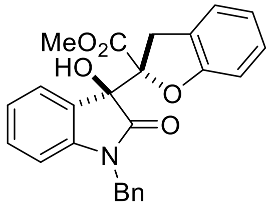	415.14	-7.0634	22.193

B4	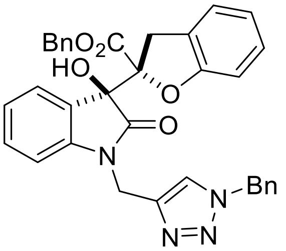	572.21	-7.6616	-13.588
B5	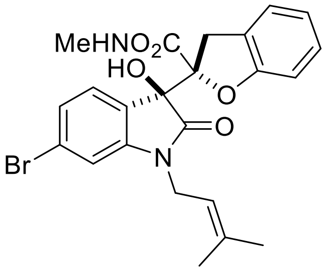	486.08	-8.0739	-38.416
B6	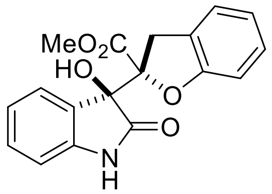	325.10	-7.3055	1.328
C1	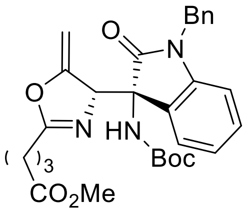	519.24	-7.4792	-1.707
C2	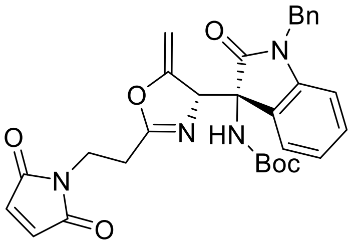	542.22	-7.4703	4.033
C3	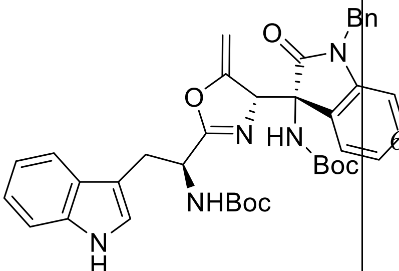	677.32	-8.1269	40.149
**C4**	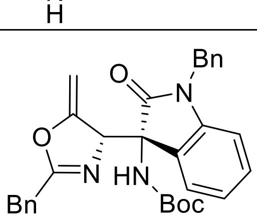	509.23	-8.4639	66.722

C5	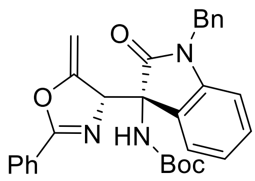	495.22	-7.2844	13.377
C7	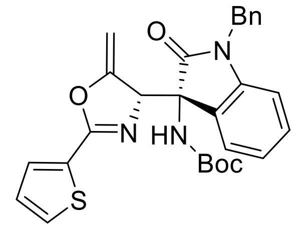	501.17	-7.3038	10.075
C8	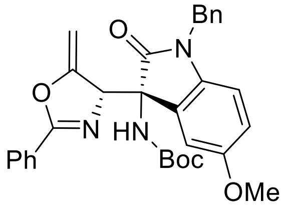	525.23	-8.1667	32.461
C9	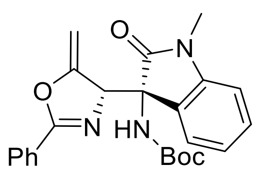	419.18	-7.6238	13.758
C10	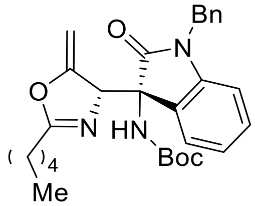	489.26	-7.8564	56.193
C11	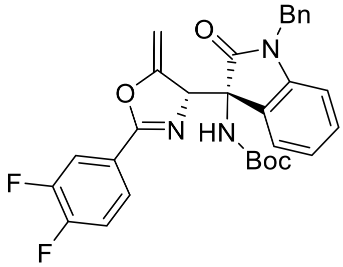	531.20	-7.7143	45.801
C12	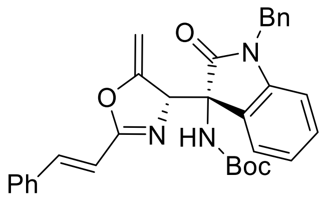	521.23	-7.8891	30.866
C13	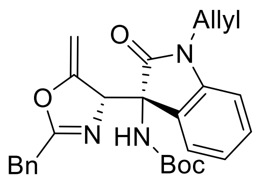	459.22	-7.7607	32.189

C14	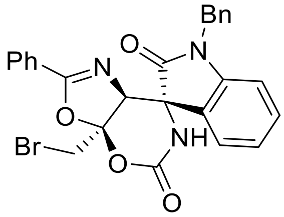	517.26	-8.6249	25.295
C15	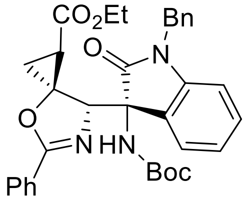	581.25	-7.1332	11.07
C16	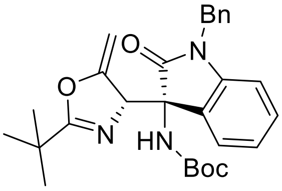	475.25	-7.3524	0.149
D1	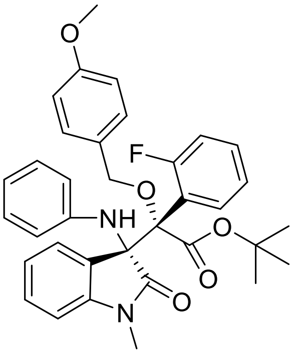	582.25	-6.5390	47.641
D2	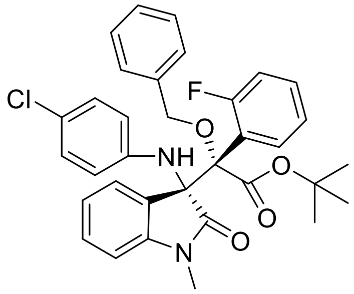	586.20	-6.0206	29.960
D3	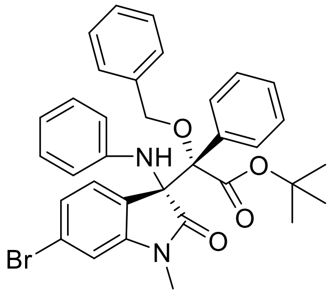	612.16	-6.0249	7.992

D4	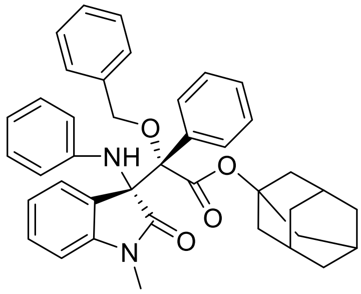	612.30	-6.2456	15.929
D5	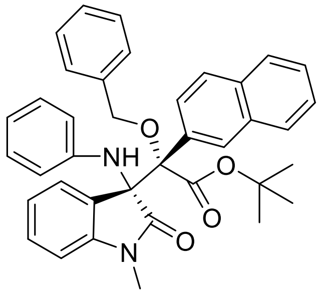	584.27	-6.2997	-59.156

6. Cell calcium imaging assay

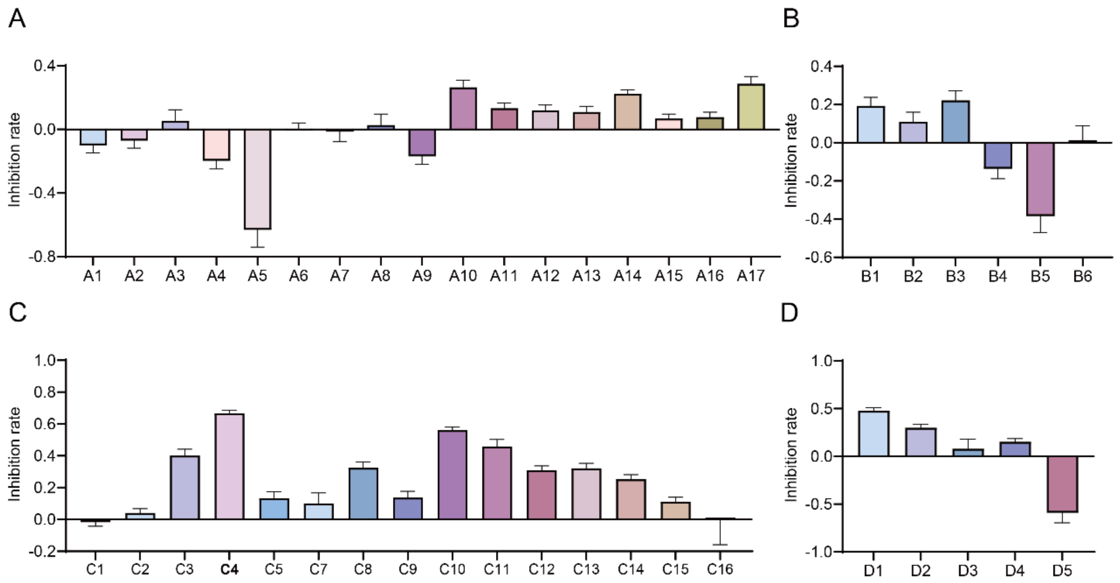

Supplementary Figure 10. Effects of compounds on VTD-evoked activity of DRG neurons. Bar graphs of average peak response of rat DRG neurons evoked by 30 µM veratridine. Neurons were incubated overnight with 0.1% DMSO or compound at 50 µM concentration. All data are presents as mean ± SEM, n ≥ 200 neurons from 2 - 3 separate coverslips.

	0.1% DMSO	C4-20.0μM
Activation		
$V_{1/2}$	-36.9 ± 0.9	-28.3 ± 0.6 (****P < 0.0001)
k	-4.9 ± 0.8	-4.7 ± 0.5 (P = 0.8861)
Inactivation		
$V_{1/2}$	-43.0 ± 1.6	-54.6 ± 2.1 (***P = 0.0002)
k	-12.2 ± 1.5	-13.8 ± 2.0 (P = 0.5093)

	0.1% DMSO	C4-1.0μM	C4-20.0μM	C4-50.0μM
Activation				
$V_{1/2}$	-19.1 ± 1.3	-18.0 ± 0.6 (P = 0.836)	-15.2 ± 1.2 (P = 0.1045)	-0.5 ± 10.9 (P = 0.2995)
k	-6.6 ± 1.1	-3.0 ± 0.5 (*P = 0.024)	-6.2 ± 1.0 (P = 0.9911)	-9.9 ± 3.8 (P = 0.786)
Inactivation				
$V_{1/2}$	-22.7 ± 1.3	-24.3 ± 0.5 (****P < 0.0001)	-32.7 ± 0.7 (P = 0.8671)	-36.9 ± 2.5 (*P = 0.0349)
k	-6.5 ± 0.7	-4.2 ± 0.4 (*P = 0.0276)	-5.2 ± 0.6 (P = 0.4719)	-6.1 ± 1.3 (P = 0.9909)

	0.1% DMSO	C4-0.1μM	C4-1.0μM	C4-20.0μM	C4-50.0μM
Activation					
$V_{1/2}$	-22.5 ± 0.9	-21.9 ± 0.9 (P = 0.9759)	-12.6 ± 0.7 (****P < 0.0001)	-10.6 ± 2.5 (****P = 0.0031)	-4.1 ± 2.8 (***P = 0.0002)
k	-19.4 ± 2.5	-18.8 ± 3.3 (P = 0.9998)	-27.3 ± 6.0 (P = 0.6458)	-15.0 ± 2.0 (P = 0.5291)	-10.2 ± 1.2 (*P = 0.0138)
Inactivation					
$V_{1/2}$	-65.8 ± 2.6	-88.6 ± 5.1 (**P = 0.0074)	-91.3 ± 10.6 (P = 0.1423)	-85.5 ± 2.7 (***P = 0.0001)	-84.0 ± 1.4 (****P < 0.0001)
k	-19.4 ± 2.5	-18.8 ± 3.3 (P = 0.9998)	-27.3 ± 6.0 (P = 0.6458)	-15.0 ± 2.0 (P = 0.5291)	-10.2 ± 1.2 (*P = 0.0138)

	0.1% DMSO	C4-0.1μM	C4-1.0μM	C4-2.0μM	C4-5.0μM	C4-10.0μM	C4-15.0μM
Activation							
$V_{1/2}$	-36.4 ± 1.0	-32.6 ± 1.4 (P = 0.2134)	-28.4 ± 0.8 (****P < 0.0001)	-22.5 ± 1.0 (****P < 0.0001)	-15.7 ± 1.1 (****P < 0.0001)	-16.0 ± 0.7 (****P < 0.0001)	-12.6 ± 0.7 (****P < 0.0001)
k	-5.0 ± 0.8	-6.7 ± 1.3 (P = 0.8559)	-5.0 ± 0.7 (P > 0.9999)	-6.0 ± 0.9 (P = 0.9533)	-7.7 ± 0.9 (P = 0.1951)	-6.1 ± 0.6 (P = 0.86)	-6.8 ± 0.5 (P = 0.4281)
Inactivation							
$V_{1/2}$	-64.0 ± 1.5	-74.0 ± 2.2 (**P = 0.0099)	-74.8 ± 2.1 (**P = 0.0035)	-74.0 ± 1.8 (*P = 0.0135)	-71.7 ± 2.3 (P = 0.0684)	-69.4 ± 2.7 (P = 0.4397)	-76.7 ± 0.6 (****P < 0.0001)
k	-16.3 ± 1.5	-15.3 ± 1.9 (P = 0.9986)	-14.3 ± 1.8 (P = 0.9331)	-12.5 ± 1.6 (P = 0.45)	-15.0 ± 2.1 (P = 0.9957)	-14.3 ± 2.4 (P = 0.9691)	-6.0 ± 0.5 (****P < 0.0001)

Supplementary Table 4. Values are means ± SEM calculated from fits of the data from the indicated number of individual cells to the Boltzmann equation; $V_{1/2}$, midpoint potential (mV) for voltage-dependent activation or inactivation; k, slope factor. Significantly different from the value for control (*$P < 0.05$, ** $P < 0.01$, **** $P < 0.0001$, Student's t-test).

7. The effect of C4 on hERG channel in whole-cell patch-clamp recording

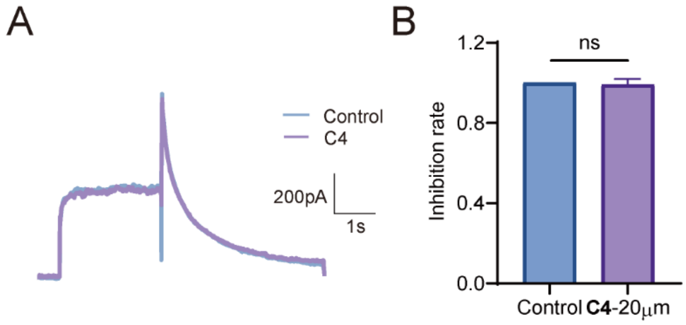

Supplementary Figure 11. The effect of **C4** on hERG channel in whole-cell patch-clamp recording (A) Representative traces of hERG currents from hERG-transfected cells. Blue trace represents the basic current without drug stimulation. Purple trace represents the current after a 10min long perfusion period of 20 μM **C4**. (**B)** A summary for hERG current inhibition of about 0.97% ± 2.9% by 20 μM **C4**. Unpaired *t*-test, $P > 0.9999$, $n = 4$.

8. The effect of 20µM C4 on Nav1.1-Nav1.4, Nav1.6 transfected HEK293 cells in whole-cell patch-clamp recording

**Inhibition rate %**	
Nav1.1	8.63% ± 0.73%
Nav1.2	9.24% ± 5.10%
Nav1.3	27.94% ± 1.18%
Nav1.4	16.02% ± 0.04%
Nav1.6	7.71% ± 1.19%

Supplementary Table 5. Under acute conditions, the effects of high concentration of **C4** on the currents of Nav1.1, Nav1.2, Nav1.3, Nav1.4, and Nav1.6 were investigated. The table represents the inhibition rates on ion channel currents before and after a continuous perfusion of 20 µM **C4** for 10 minutes. Values represent means ± SEM.

9. Evaluation of the temporal effect of 20µM C4 on Nav1.7-transfected HEK293 cells in whole-cell patch-clamp recording

A

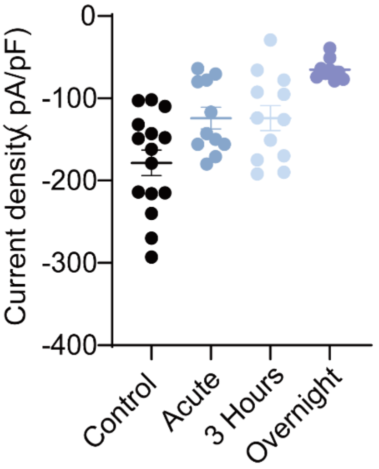

B

Inhibition rate %	
Acute (10 min)	30.4% ± 7.37%
3 Hours	30.5% ± 8.56%
Overnight	63.4% ± 2.39%

Supplementary Figure 12. The temporal effect of 20µM **C4** on Nav1.7-transfected HEK293 cells (A) Time course analysis of peak current density treated with 20µM **C4** (Control, -178.44 ± 15.6; Acute, -124.1 ± 13.2; 3 hours, -124.0 ± 13.2; Overnight, -65.2 ± 4.3). **(B)** A summary inhibition rate at different time course on Nav1.7 current by 20 µM **C4**. Values represent means ± SEM.

Supplementary References

1. Zhou K*, et al.* Highly diastereoselective synthesis of vicinal diamines via a Rh-catalyzed three-component reaction of diazo compounds with diarylmethanimines and ketimines. *Org. Chem. Front.* **8**, 2997-3003 (2021).

2. Dong G, Bao M, Xie X, Jia S, Hu W, Xu X. Asymmetric Allylation by Chiral Organocatalyst-Promoted Formal Hetero-Ene Reactions of Alkylgold Intermediates. *Angew. Chem. Int. Ed. Engl.* **60**, 1992-1999 (2021).

3. Kang Z, Shou J, Xing D, Hu W. Rh(II)/Ag(I)-Cocatalyzed Three-Component Reaction via SN1/SN1'-Type Trapping of Oxonium Ylide with the Nicholas Intermediate. *J. Org. Chem.* **85**, 9850-9862 (2020).

4. Zhang D, Wang X, Zhang M, Hu W. Rhodium-Catalyzed Sequential Cycloisomerization/Aldol Addition of Cyclopropene Carboxylic Acids with Isatins. *Org. Lett.* **22**, 5600-5604 (2020).

5. Gopi Krishna Reddy A*, et al.* Bronsted Acid Catalyzed Enantioselective Assembly of Spirochroman-3,3-oxindoles. *Org. Lett.* **22**, 2925-2930 (2020).

6. Kang Z, Zhang D, Xu X, Hu W. Privilege-Structure-Oriented Three-Component Asymmetric Aminomethylation: Assembly of Chiral 3-Aminomethyl Indolones. *Org. Lett.* **21**, 9878-9883 (2019).

7. Ao C*, et al.* Zinc-Catalyzed Alkyne-Carbonyl Metathesis of Ynamides with Isatins: Stereoselective Access to Fully Substituted Alkenes. *J. Org. Chem.* **84**, 15331-15342 (2019).

8. Shi T, Teng S, Wei Y, Guo X, Hu W. Synthesis of spiro[2,3-dihydrofuran-3,3′-oxindole] derivatives via a multi-component cascade reaction of α-diazo esters, water, isatins and malononitrile/ethyl cyanoacetate. *Green. Chem.* **21**, 4936-4940 (2019).

9. Che J, Gopi Krishna Reddy A, Niu L, Xing D, Hu W. Cu(I)-Catalyzed Three-Component Reaction of alpha-Diazo Amide with Terminal Alkyne and Isatin Ketimine via Electrophilic Trapping of Active Alkynoate-Copper Intermediate. *Org. Lett.* **21**, 4571-4574 (2019).

10. Jia S, Yang X, Dong G, Ao C, Jiang X, Hu W. Trapping of Zwitterionic Intermediates by Isatins and Imines: Synthesis of Benzoxazines Bearing a C4-Quaternary Stereocenter. *Org. Lett.* **21**, 4014-4018 (2019).

11. Zhang D, Kang Z, Liu J, Hu W. Metal-Dependent Umpolung Reactivity of Carbenes Derived from Cyclopropenes. *iScience.* **14**, 292-300 (2019).

12. Xiao G, Chen T, Ma C, Xing D, Hu W. Rh(II)/Chiral Phosphoric Acid-Cocatalyzed Enantioselective Synthesis of Spirooxindole-Fused Thiaindans. *Org. Lett.* **20**, 4531-4535 (2018).

13. Ma C, Chen J, Xing D, Sheng Y, Hu W. Iron catalyzed efficient synthesis of poly-functional primary amines via the direct use of ammonia. *Chem. Commun. (Camb).* **53**, 2854-2857 (2017).

14. Jia S, Lei Y, Song L, Krishna Reddy AG, Xing D, Hu W. Diastereoselective Intramolecular Aldol-Type Trapping of Zwitterionic Intermediates by Ketones for the Synthesis of Spiro[chroman-4,3′-oxindole] Derivatives. *Adv. Synth. Catal.* **359**, 58-63 (2017).

15. Jia S-K, Lei Y-B, Song L-L, Liu S-Y, Hu W-H. Enantioselective trapping of oxonium ylide intermediates by N -benzhydryl- α -imino ester: Synthesis of β -tetrasubstituted α -amino acids. *Chinese. Chem. Lett.* **28**, 213-217 (2017).

16. Dong S*, et al.* Discovery of core-structurally novel PTP1B inhibitors with specific selectivity containing oxindole-fused spirotetrahydrofurochroman by one-pot reaction. *Bioorg. Med. Chem. Lett.* **27**, 1105-1108 (2017).

17. Jia SK, Song LL, Lei YB, Gopi Krishna Reddy A, Xing D, Hu WH. A Rh(ii)-catalyzed three-component reaction of 3-diazooxindoles with N,N-disubstituted anilines and glyoxylates for the synthesis of 3-aryl-3-substituted oxindoles. *Org. Biomol. Chem.* **14**, 10157-10160 (2016).

18. Jing C, Xing D, Hu W. Catalytic Asymmetric Four-Component Reaction for the Rapid Construction of 3,3-Disubstituted 3-Indol-3'-yloxindoles. *Org. Lett.* **17**, 4336-4339 (2015).

19. Jing C, Xing D, Wang C, Hu W. Synthesis of 3-(hydroxymethyl)-3-indol-3′-yloxindoles via Rh(II)-catalyzed three-component reaction of 3-diazooxindoles, indoles and formalin. *Tetrahedron.* **71**, 3597-3602 (2015).

20. Liu X*, et al.* Enantioselective Propargylation of Oxonium Ylide with alpha-Propargylic-3-Indolymethanol: Access to Chiral Propargylic Indoles. *Org. Lett*. **24**, 1027-1032 (2022).

21. Hong K*, et al.* Diastereoselective aldol-type interception of phenolic oxonium ylides for the direct assembly of 2,2-disubstituted dihydrobenzofurans. *Org. Biomol. Chem.* **20**, 4635-4639 (2022).

22. Yang X*, et al.* Asymmetric Three-Component Reaction of Two Diazo Compounds and Hyrdroxylamine Derivatives for the Access to Chiral α-Alkoxy-β-amino-carboxylates.

ACS Catalysis **12**, 12302-12309 (2022).
