# Supplementary material for: Carbenoid-involved reactions integrated with scaffold-based screening generates a Nav1.7 inhibitor
Source: Commun Chem. 2024 Jun 12;7:135. doi: 10.1038/s42004-024-01213-3 (PMC11169417; doi:10.1038/s42004-024-01213-3)
Supplement: Supplementary file 3 — Description of Additional Supplementary Files [file 42004_2024_1213_MOESM3_ESM.pdf]

Description of Additional Supplementary Files

File name- Supplementary Data 1

File description- source data for fig.6

File name- Supplementary Data 2

File description- source data for fig.7

File name- Supplementary Data 3

File description- source data for fig.8

File name- Supplementary Data 4

File description- source data for fig.9

File name- Supplementary Data 5

File description- source data for fig.S8

File name- Supplementary Data 6

File description- source data for fig.S9

File name- Supplementary Data 7

File description- source data for fig.S10

File name- Supplementary Data 8

File description- source data for fig.S5

File name- Supplementary Data 9

File description- source data for Molecular dynamics

File name- Supplementary Data 10

File description- source data for virtual screening
