# Supplementary material for: Carbenoid-involved reactions integrated with scaffold-based screening generates a Nav1.7 inhibitor
Source: Commun Chem. 2024 Jun 12;7:135. doi: 10.1038/s42004-024-01213-3 (PMC11169417; doi:10.1038/s42004-024-01213-3)
Supplement: Supplementary file 14 — Reporting Summary [file 42004_2024_1213_MOESM14_ESM.pdf]

nature portfolio

Corresponding author(s): Song Cai, Taoda Shi, and Wenhao Hu

Last updated by author(s): 2024/04/20

Reporting Summary

Nature Portfolio wishes to improve the reproducibility of the work that we publish. This form provides structure for consistency and transparency in reporting. For further information on Nature Portfolio policies, see our Editorial Policies and the Editorial Policy Checklist.

Statistics

For all statistical analyses, confirm that the following items are present in the figure legend, table legend, main text, or Methods section.

n/a	Confirmed	
☐	☒	The exact sample size (n) for each experimental group/condition, given as a discrete number and unit of measurement
☐	☒	A statement on whether measurements were taken from distinct samples or whether the same sample was measured repeatedly
☒	☐	The statistical test(s) used AND whether they are one- or two-sided<br>*Only common tests should be described solely by name; describe more complex techniques in the Methods section.*
☐	☒	A description of all covariates tested
☐	☒	A description of any assumptions or corrections, such as tests of normality and adjustment for multiple comparisons
☐	☒	A full description of the statistical parameters including central tendency (e.g. means) or other basic estimates (e.g. regression coefficient) AND variation (e.g. standard deviation) or associated estimates of uncertainty (e.g. confidence intervals)
☒	☐	For null hypothesis testing, the test statistic (e.g. F, t, r) with confidence intervals, effect sizes, degrees of freedom and P value noted<br>*Give P values as exact values whenever suitable.*
☒	☐	For Bayesian analysis, information on the choice of priors and Markov chain Monte Carlo settings
☒	☐	For hierarchical and complex designs, identification of the appropriate level for tests and full reporting of outcomes
☒	☐	Estimates of effect sizes (e.g. Cohen's d, Pearson's r), indicating how they were calculated

Our web collection on statistics for biologists contains articles on many of the points above.

Software and code

Policy information about availability of computer code

Data collection	The OREAL library was created by open-soure tool RDkit, the code is avaliable at website: https://rdkit.org/<br>The molecular Molecular Dynamic simulation was taken under by free and open-source software Gromacs.
Data analysis	1. The NMR data recorded on Brucker Avance 400 MHz or Ascend 500 MHz spectrometers, and were processed by MestRenova 10.0.0<br>2. Molecular docking and virtual screening was peformed by MOE(MOE (The Molecular Operating Environment, Chemical Computing Group Inc.) and the results were analyzed by PyMol 2.0 and Schrödinger software (Release 2019-2, Schrödinger LLC, New York, NY, 2019)<br>3. The PCA analysis was taken by Datawarrior. (https://openmolecules.org/datawarrior/)<br>4. The PMI analysis of OREAL library and commercial library was taken by open-soure tool RDkit. (https://rdkit.org/)

For manuscripts utilizing custom algorithms or software that are central to the research but not yet described in published literature, software must be made available to editors and reviewers. We strongly encourage code deposition in a community repository (e.g. GitHub). See the Nature Portfolio guidelines for submitting code & software for further information.

Data

Policy information about availability of data

All manuscripts must include a data availability statement. This statement should provide the following information, where applicable:

- Accession codes, unique identifiers, or web links for publicly available datasets
- A description of any restrictions on data availability
- For clinical datasets or third party data, please ensure that the statement adheres to our policy

1. The OREAL library of the synthetic methodology is avaliable at http://www.sysu-sps-compound.com.
2. NMR, HMRS, PCA analysis and Biological activity data ,IC50s, virtual screening, molecular docking model and MD simulation are avaliable in supplementary file.
3. Nav1.7 co-crystal structure was free-downloaded from https://www.rcsb.org/ (PDBID: 5EK0)

Human research participants

Policy information about studies involving human research participants and Sex and Gender in Research.

Reporting on sex and gender	N/A
Population characteristics	N/A
Recruitment	N/A
Ethics oversight	N/A

Note that full information on the approval of the study protocol must also be provided in the manuscript.

Field-specific reporting

Please select the one below that is the best fit for your research. If you are not sure, read the appropriate sections before making your selection.

☒ Life sciences ☐ Behavioural & social sciences ☐ Ecological, evolutionary & environmental sciences

For a reference copy of the document with all sections, see nature.com/documents/nr-reporting-summary-flat.pdf

Life sciences study design

All studies must disclose on these points even when the disclosure is negative.

Sample size	For each experiment, sample sizes were at least n=8 independent biological replicates in patch recording. In behavioral studies, n=6, while in calcium imaging experiments, a minimum of n=100 is required. Additionally, annotations are provided in each experiment.
Data exclusions	N/A
Replication	Information about replicate numbers are detailed in the figure legends and supplementary inforamtion
Randomization	In all experiments samples were randomly allocated to groups.
Blinding	For animal experiment the investigators where blinded to the group allocation during data collection. For other experiments blinding was not considered relevant.

Reporting for specific materials, systems and methods

We require information from authors about some types of materials, experimental systems and methods used in many studies. Here, indicate whether each material, system or method listed is relevant to your study. If you are not sure if a list item applies to your research, read the appropriate section before selecting a response.

Materials & experimental systems

n/a	Involved in the study
☒	☐ Antibodies
☐	☒ Eukaryotic cell lines
☒	☐ Palaeontology and archaeology
☐	☒ Animals and other organisms
☒	☐ Clinical data
☒	☐ Dual use research of concern

Methods

n/a	Involved in the study
☒	☐ ChIP-seq
☒	☐ Flow cytometry
☒	☐ MRI-based neuroimaging

Eukaryotic cell lines

Policy information about cell lines and Sex and Gender in Research

Cell line source(s)	The cells are sourced from BeiOuTes Technology and the Shanghai Institute of Life Sciences, Chinese Academy of Sciences.
Authentication	N/A
Mycoplasma contamination	N/A
Commonly misidentified lines (See ICLAC register)	No commonly misidentified lines were used in this study.

Animals and other research organisms

Policy information about studies involving animals; ARRIVE guidelines recommended for reporting animal research, and Sex and Gender in Research

Laboratory animals	Adult male Sprague-Dawley rats (came from Beijing Viton Lever)
Wild animals	No wild animals were used in this study.
Reporting on sex	This experiment exclusively used male rats, in accordance with the majority of literature which does not differentiate based on sex.
Field-collected samples	No field collected samples were in this experiment.
Ethics oversight	Experiments were conducted in accordance with Institutional Guidelines under acquired permission from the local Ethical Committee on Animal Experimentation

Note that full information on the approval of the study protocol must also be provided in the manuscript.
